# Common and distinct roles of amygdala subregional functional connectivity in non-motor symptoms of Parkinson’s disease

**DOI:** 10.1038/s41531-023-00469-1

**Published:** 2023-02-17

**Authors:** Junling Wang, Lianglong Sun, Lili Chen, Junyan Sun, Yapei Xie, Dezheng Tian, Linlin Gao, Dongling Zhang, Mingrui Xia, Tao Wu

**Affiliations:** 1 Center for Movement Disorders, Department of Neurology, Beijing Tiantan Hospital, Capital Medical University, Beijing, 100070 China; 2 State Key Laboratory of Cognitive Neuroscience and Learning, Beijing Normal University, Beijing, 100091 China; 3 Beijing Key Laboratory of Brain Imaging and Connectomics, Beijing Normal University, Beijing, 100091 China; 4 IDG/McGovern Institute for Brain Research, Beijing Normal University, Beijing, 100091 China; 5 Department of General Medicine, Tianjin Union Medical Center, Tianjin, 300122 China

**Keywords:** Parkinson's disease, Parkinson's disease

## Abstract

Neuroimaging studies suggest a pivotal role of amygdala dysfunction in non-motor symptoms (NMS) of Parkinson’s disease (PD). However, the relationship between amygdala subregions (the centromedial (CMA), basolateral (BLA) and superficial amygdala (SFA)) and NMS has not been delineated. We used resting-state functional MRI to examine the PD-related alterations in functional connectivity for amygdala subregions. The left three subregions and right BLA exhibited between-group differences, and were commonly hypo-connected with the frontal, temporal, insular cortex, and putamen in PD. Each subregion displayed distinct hypoconnectivity with the limbic systems. Partial least-squares analysis revealed distinct amygdala subregional involvement in diverse NMS. Hypo-connectivity of all four subregions was associated with emotion, pain, olfaction, and cognition. Hypo-connectivity of the left SFA was associated with sleepiness. Our findings highlight the hypofunction of the amygdala subregions in PD and their preliminary associations with NMS, providing new insights into the pathogenesis of NMS.

## Introduction

Parkinson’s disease (PD) is the second most common neurodegenerative disorder, characterized by the loss of dopamine cells in the substantia nigra and formation of intracellular inclusions (Lewy bodies) containing aggregates of α-synuclein^1^. Lewy bodies are found in subcortical and cortical regions as disease progresses, causing basal ganglia-cortical circuit dysfunction and multiple neurotransmitter deficits that result in a wide range of motor and non-motor symptoms (NMS)^2^. The NMS of PD, such as hyposmia, sleep, mood, cognitive, and autonomic disturbances, dominate the prodromal phase of PD^3^ and become increasingly prevalent during disease progression, greatly affecting the quality of life and severity of disability of patients^4^. Furthermore, a multicenter survey revealed that 98.6% of PD patients have NMS^5^. However, the underlying pathophysiological mechanisms of NMS in PD remain unclear, which impedes the development of effective therapeutic strategies.

Advances in resting-state functional MRI (rs-fMRI) have provided a powerful technique for examining the intrinsic characteristics of functional networks, which has been widely applied to study the neurobiological mechanisms underlying NMS in PD. Recent rs-fMRI studies revealed widespread abnormalities in functional connectivity (FC) within fear and cortico-striato-thalamocortical limbic circuits in PD, and FC abnormalities in these circuits are significantly associated with NMS^6–8^. In particular, amygdala showed disrupted functional connectivity with extensive brain regions of the fear circuit, including the prefrontal cortex, medial temporal cortex, basal ganglia, and limbic system^8,9^. The hypo-connectivity of these regions were involved in diverse range of NMS, such as emotional disturbances^10^, olfactory and cognitive dysfunction^11^, impulsive-control disorder (ICD)^12^, and sleep disturbance^13^. Anatomically, the amygdala is divided into three main subregions based on differences in cytoarchitecture and receptor architecture: the basolateral complex (BLA), centromedial complex (CMA), and superficial cortex-like complex amygdala (SFA)^14^. FC analysis confirmed that common and distinct connectivity patterns exist across these subregions in healthy adults^15^. All subregions exhibit strong connections with the prefrontal cortex (PFC) and insular cortex. The BLA is primarily connected to the temporal and frontal regions, whereas the CMA is mainly connected to the striatum, and the SFA has extensive connections to the limbic cuicuit^16^. Furthermore, amygdala subregions have common and unique connectivity abnormalities in neuropsychiatric disorders including psychopathy^17^, post-traumatic stress disorder^18^, and attention deficit hyperactivity disorders^19^. However, the common and unique FC changes of amygdala subregions in PD are unknown, and it is unclear how functional abnormalities of amygdala subregions contribute to the pathophysiology of diverse aspects of NMS in PD.

The aims of this study were to (1) characterize FC abnormalities of amygdala subregions, and (2) comprehensively assess how amygdala subregional dysfunctions contribute to the clinical phenotype of NMS in PD. We hypothesized that amygdala subregions would exhibit common and distinct hypo-connectivity in PD, and hypo-connectivity of amygdala subregions would correlate to the diversity of NMS.

## Results

### Demographic, clinical, and morphological parameters

In total, 115 patients with PD and 78 healthy controls (HCs) were included in this study. No significant differences were observed regarding age, sex, or education level between the HC and PD groups. Patients with PD had significantly worse smell, cognition, sleep, emotional, and autonomic scores than HCs (Table 1). Gray matter (GM) volume was significantly lower in the PD group than in the HC group, but there was no significant difference in the volume of the amygdala subregions between the two groups (Supplementary Table 1).

**Table 1:** Demographic and clinical information of the participants.

Characteristics	PD group	HC group	*P* value
Age, years	59.50 ± 9.4 (41–80)	60.68 ± 8.8 (41–80)	0.384
Gender, male/female	59/56	36/42	0.482
Education, years	12 [9, 16] (5–22)	12 [9, 16] (6–20)	0.404
**Motor symptoms**			
Disease duration, years	2.5 [1.17, 4.75] (0.08–21)	–	–
Hoehn-Yahr stage	2 [1, 2] (1–3)	–	–
MDS-UPDRS III	25.73 ± 12.08 (6–56)	–	–
LEDD, mg	300 [0, 450] (0–1462.5)	–	–
**Non-motor symptoms**			
UPSIT	25 [18, 31] (5–40)	36 [35, 37] (30–40)	**<0.001**
MoCA	25 [22, 28] (12–30)	27 [26, 28] (26–30)	**<0.001**
RBD-SQ	4 [2, 7] (1–13)	3 [2, 4] (1–4)	**<0.001**
Epworth sleepiness scale	5 [2, 8] (0–21)	4 [2, 6] (0–10)	0.114
QUIP	0 [0, 0] (0–3)	0 [0, 0] (0–0)	**<0.001**
GDS-15	2 [1, 4] (0–10)	0 [0, 0] (0–3)	**0.005**
STAI (state anxiety)	33 [26, 36] (20–58)	26 [22, 27] (20–35)	**<0.001**
STAI (trait anxiety)	37 [28, 44] (20–63)	28 [25, 30] (20–34)	**<0.001**
Apathy (MDS UPDRS I-1.5)	0 [0, 1] (0–3)	0 [0, 0] (0–1)^a^	**<0.001**
Hallucination (UPDRS I-1.2)	0 [0, 0] (0–2)	0 [0, 0] (0–0)^a^	**0.048**
Pain (UPDRS I-1.9)	0 [0, 1] (0–4)	0 [0, 0] (0–1)^a^	**0.002**
Urinary disorder (UPDRS I-1.10)	1 [0, 1] (0–3)	0 [0, 0] (0-1)^a^	**0.001**
Constipation (UPDRS I-1.11)	0 [0, 1] (0–4)	0 [0, 0] (0–1)^a^	**<0.001**
OH (UPDRS I-1.12)	0 [0, 1] (0–3)	0 [0, 0] (0–1)^a^	0.093
Fatigue (UPDRS I-1.13)	1 [0, 1] (0–4)	0 [0, 0] (0–1)^a^	**<0.001**
Drooling (UPDRS II-2.2)	0 [0, 2] (0–3)	0 [0, 0] (0–0)^a^	**<0.001**
Swallowing (UPDRS II-2.3)	0 [0, 0] (0–3)	0 [0, 0] (0–0)^a^	**0.020**

Values are presented as mean ± standard deviation (SD) or median [quartile] and (min-max). Bold values indicate significant differences between two groups.

*PD* Parkinson’s disease, *HC* healthy controls, *LEDD* levodopa-equivalent daily dose, *GDS-15* 15-item Geriatric Depression Scale, *UPSIT* University of Pennsylvania Smell Identification Test, *MoCA* Montreal cognitive assessment, *RBD-SQ* rapid eye movement sleep behavior disorder screening questionnaire, *QUIP* Parkinson’s Disease Impulsive-Compulsive Disorders Questionnaire-current short, *STAI* The State-Trait Anxiety Inventory, *OH* orthostatic hypotension.

^a^30 HC rated MDS UPDRS I and II.

### Functional connectivity of amygdala subregions in PD

#### Functional connectivity profile of amygdala subregions

**Fig. 1:**
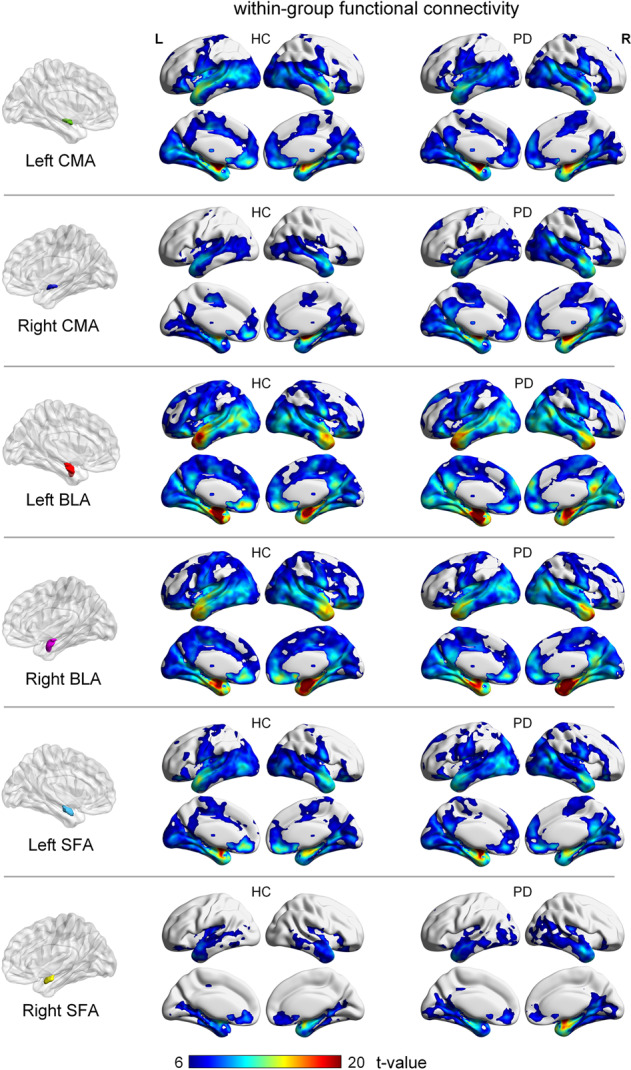
Within-group functional connectivity (FC) of amygdala subregions. Results thresholded at FWE-corrected voxel-level *P* < 0.05 (cluster size>10 voxels) in MNI152 space.

Within-group functional connectivity analysis revealed that the general FC pattern of the amygdala subregions were retained in patients with PD relative to the HCs. Specifically, the six amygdala subregions commonly had positive functional connectivity with several cortical regions in both HC and PD groups, including the bilateral medial PFC (mPFC), medial and lateral temporal cortices, cingulate cortex, and insula (Fig. 1). Each amygdala subregion also exhibited specific connections in both HC and PD groups, for example, the bilateral BLA was more extensively connected to the frontal and parietal cortex compared to the CMA and SFA; the right SFA had fewer connections with the frontal and limbic lobes than the left SFA.

### Abnormal functional connectivity of amygdala subregions in PD

**Fig. 2:**
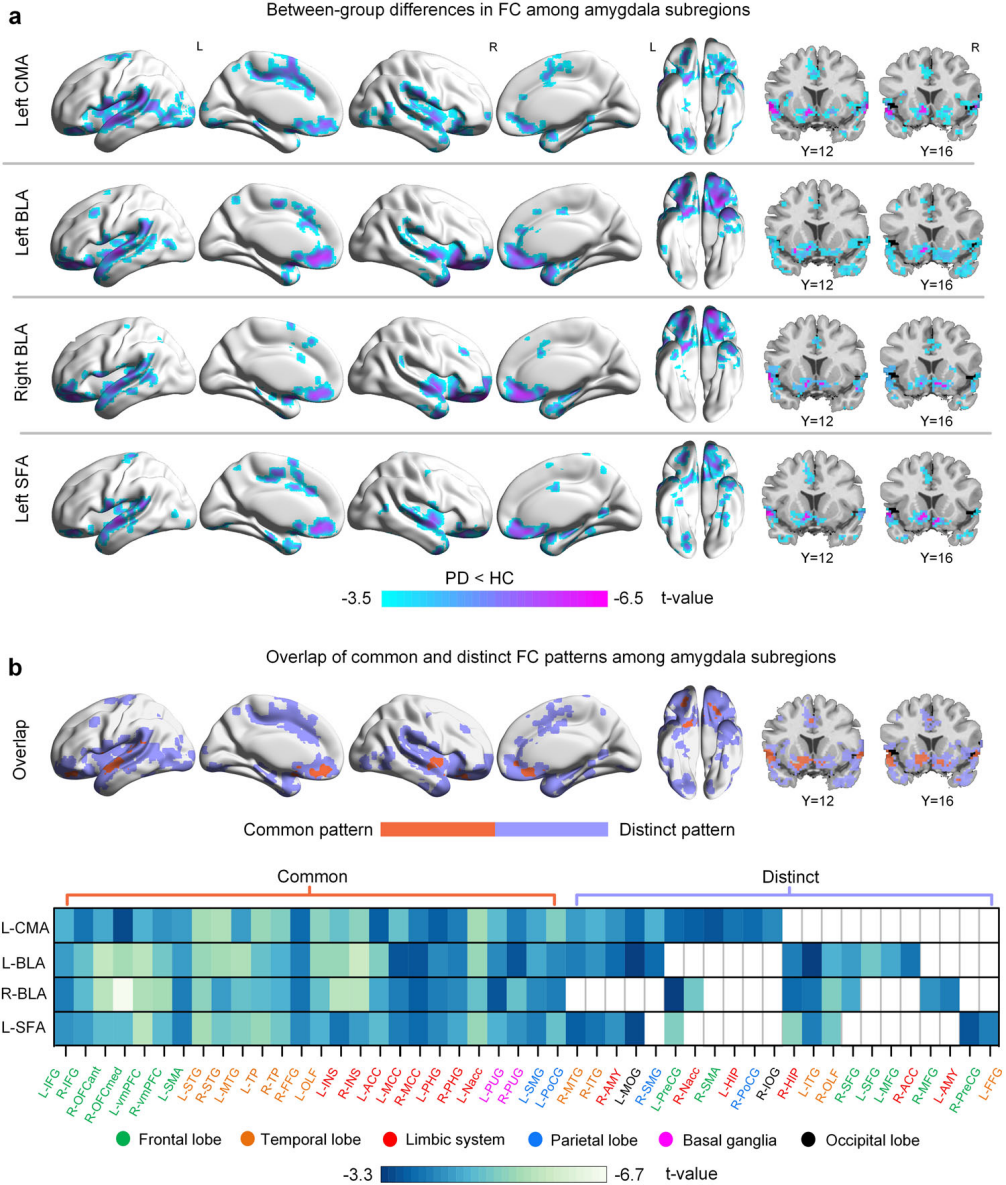
Between-group differences in functional connectivity of amygdala subregions. **a** Significant group differences in FC for each amygdala subregion. Group-difference FC maps are thresholded at FDR-corrected voxel-level *P* < 0.05 (MNI 152 space, cluster size ≥10 voxels). **b** Spatial similarities and differences of FC maps among amygdala subregions. IFG Inferior frontal gyrus, OFCant Anterior orbital gyrus, OFCmed Medial orbital gyrus, vmPFC Ventromedial prefrontal cortex, SMA Supplementary motor area, STG Superior temporal gyrus, MTG Middle temporal gyrus, TP Temporal pole, FFG Fusiform gyrus, OLF Olfactory cortex, INS Insula, ACC Anterior cingulate cortex, MCC Middle cingulate cortex, PHG Parahippocampal gyrus, Nacc Nucleus accumbens, PUG Putamen, SMG SupraMarginal gyrus, PoCG Postcentral gyrus, ITG Inferior temporal gyrus, AMY Amygdala, MOG Middle occipital gyrus, PreCG Precentral gyrus, HIP Hippocampus, IOG Inferior occipital gyrus, SFG Superior frontal gyrus, MFG Middle frontal gyrus.

Statistical between-group comparisons showed that, only four subregions (the left CMA, left BLA, left SFA, and right BLA) presented statistically significant FC differences in the PD group compared with HC (the right CMA and right SFA presented no significant FC differences between the two groups) (Fig. 2a). All amygdala subregion (left CMA, left BLA, right BLA, and left SFA) had significantly lower functional connectivity with the bilateral ventral medial PFC (vmPFC), orbital frontal gyrus (OFC), inferior frontal gyrus (IFG), superior and middle temporal gyri (STG/MTG), temporal pole (TP), olfactory cortex, insula, anterior and middle cingulate cortices (ACC/MCC), parahippocampus, putamen, and left supplementary motor area (SMA) in the PD group. Distinct PD-related hypo-connectivity patterns were also observed. The left CMA displayed lower functional connectivity with the left hippocampus (HIP), right postcentral gyrus (PoCG) and SMA. The left BLA showed lower connectivity with bilateral superior frontal gyri. The right BLA showed lower connectivity with the left amygdala, and the left SFA exhibited lower connectivity with the left fusiform gyrus and right precentral gyrus (PreCG) (FDR-corrected voxel level, *P* < 0.05; k ≥ 10 voxels) (Fig. 2b and Supplementary Table 2).

### The relationship between amygdala subregional FC and clinical signature in PD

**Fig. 3:**
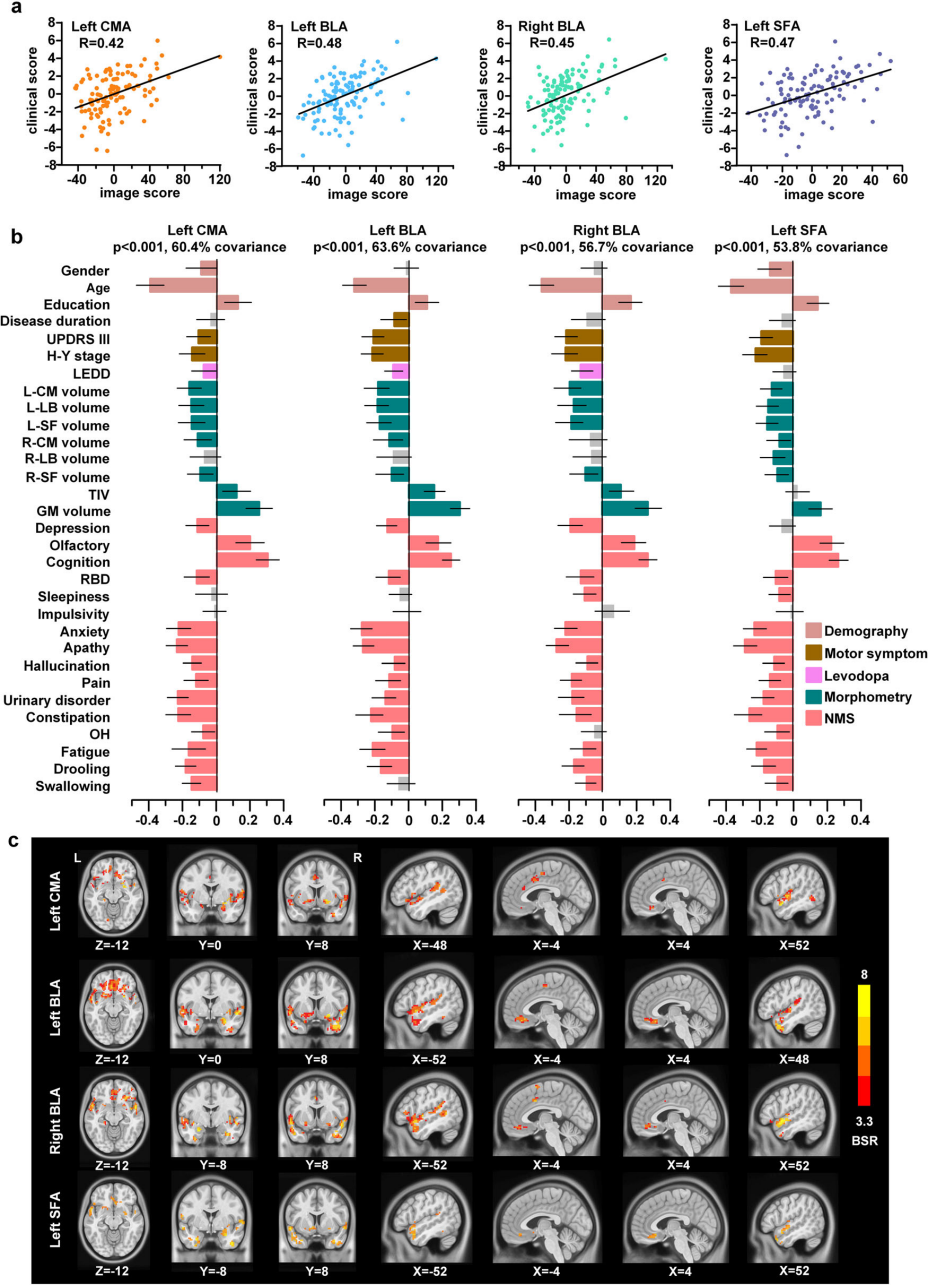
Amygdala subregion-specific comprehensive clinical-functional signatures in PD. **a** Brain scores and behavior scores of LV-1 demonstrate significant correlations in each subregion. Individuals who exhibited the FC pattern in **c** tend to exhibit corresponding behavioral phenotypes in **b**. The colors in **a** represent different subregions. **b** Subregion-specific clinical features. The correlation of each subregion to each clinical feature is shown using correlation coefficients. Error bars indicate bootstrap estimated 95% confidence intervals of correlation strength. The colors in **b** represent the different categories of clinical symptoms. **c** Subregion-specific FC maps associate with clinical features. Patients who exhibit these FC patterns tend to score worse regarding clinical symptoms. The contribution of hypo-connectivity voxels on clinical features is shown using bootstrap ratio (BSR), thresholding at BSR > 3.3 (*P* < 0.001, MNI 152 space). A larger BSR represents a greater contribution to the LV that represent the relationship between hypo-connectivity features and NMS. LEDD levodopa-equivalent daily dose, TIV total intracranial volume, OH orthostatic hypotension.

Only four subregions (left CMA, left BLA, left SFA, and right BLA) showed statistically significant FC differences between the PD and HC groups; therefore, these four subregions were used to perform the following correlation analysis. Partial least-squares (PLS) analysis identified one significant latent variable (LV) for the left CMA (62.7% covariance, *P* < 0.001), one LV for the left BLA (64.0% covariance, *P* < 0.001), one LV for the right BLA (56.9% covariance, *P* < 0.001), and one LV for the left SFA (55.5% covariance, *P* < 0.001) (Supplementary Fig. 1). Significant correlations were found in the PLS scores from FC (brain score) and clinical symptoms (behavior score) in each subregion (Fig. 3a). Figure 3b illustrates that all amygdala subregions were associated with demographic characteristics (gender, age, and education), motor symptoms (disease duration, MDS Unified Parkinson’s Disease Rating Scale Part III (MDS-UPDRS-III), and H-Y stage), dopaminergic drugs, brain volumes (total intracranial volume (TIV), GM, and amygdala), mood disorders, hyposmia, cognitive deficits, pain, and autonomic dysfunction. These symptoms were mainly associated with connectivity deficits in the vmPFC, OFC, temporal lobe, putamen, and limbic system, such as the contralateral amygdala, nucleus accumbens (Nac), and cingulate cortices (bootstrap ratio (BSR) > 3.3) (Fig. 3c, Supplementary Table 3, and Supplementary Table 4).

**Fig. 4:**
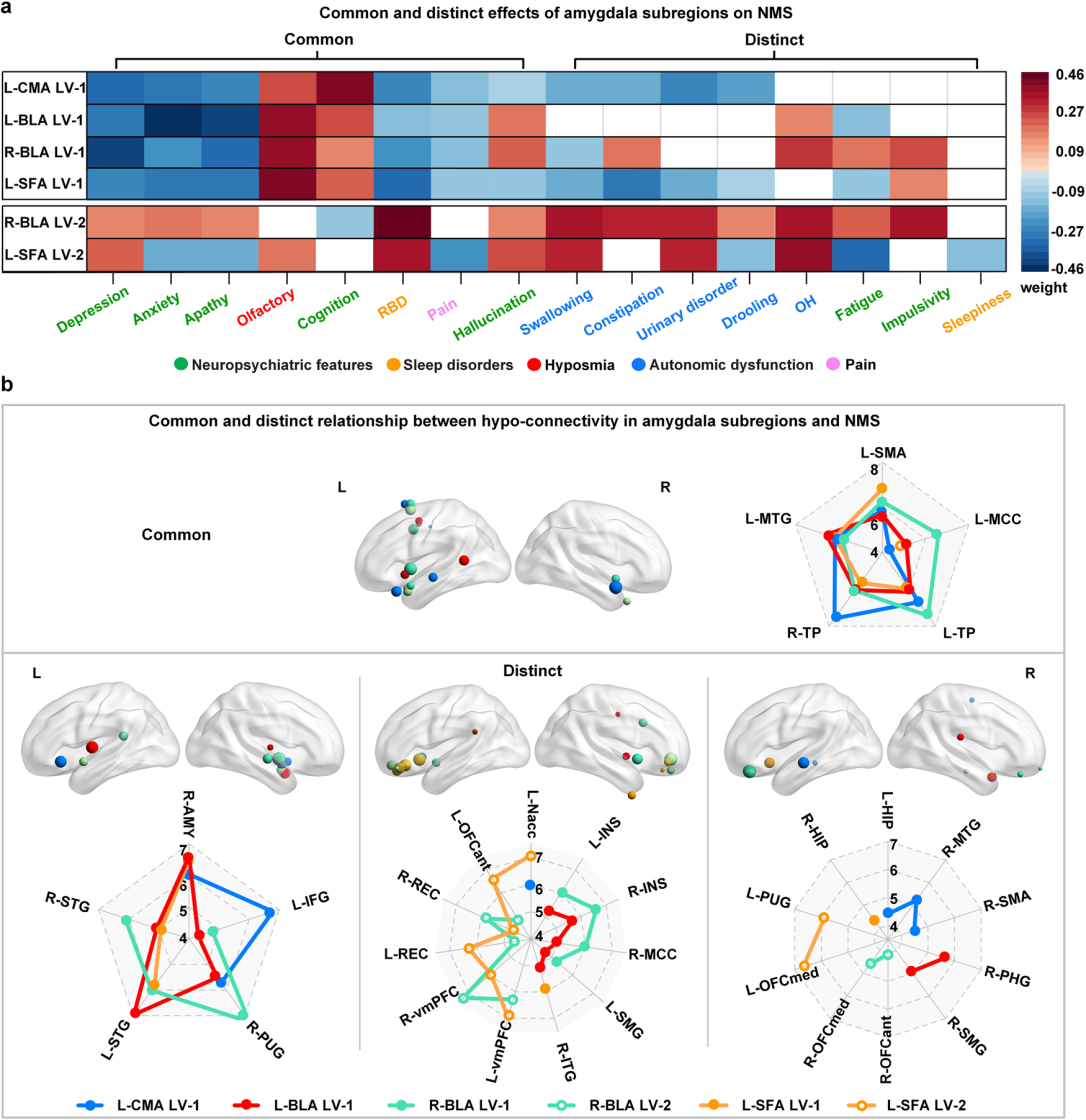
Common and distinct relationship between hypo-connectivity in amygdala subregions with NMS. **a** The correlations of amygdala subregions to different NMS phenotypes. **b** Summary of spatial correlation of hypo-connectivity of amygdala subregions to NMS. The size of the spheres in the brain indicates the bootstrap ratio (BSR) values, larger spheres represent the greater contribution to the LV that represent the relationship between hypo-connectivity features and NMS. Brain maps show the location of brain regions, while radar maps show the BSR value of brain regions. The commonly brain region map (top left) means that each region in this map exhibits hypo-connectivity with all four amygdala subregions. While distinct brain map mean that the region exhibits hypo-connectivity with only one or some of the four sub-regions.

Moreover, as demographic characteristics (e.g., gender, age, and education), clinical features (e.g., disease duration, UPDRS III, and H-Y stage), medication (levodopa equivalent daily dose (LEDD)), and whole-brain and amygdala volumes may be associated with the severity of NMS and the dysfunction of the amygdala subregions, we used multiple linear regression to regressed off these confounding variables in both NMS scores and FC maps and re-performed the PLS analysis to further explore the role of the amygdala in NMS of PD (Supplementary Figs. 2, 3, and Supplementary Table 5). Common and distinct roles of the amygdala subregions in NMS were observed in NMS-specific PLS results. All four subregions exhibited common roles in emotional disturbances (depression, anxiety, apathy), hyposmia, cognitive deficits, rapid eye movement sleep behavior disorder (RBD), pain, and hallucinations (Fig. 4a, Supplementary Table 6). Furthermore, different NMS phenotypes were associated with distinct subregions. Autonomic dysfunctions, such as urinary disorders, constipation, drooling, and difficulty swallowing were mainly associated with hypo-connectivity of the left CMA, right BLA, and left SFA. Fatigue and orthostatic hypotension were associated with bilateral BLA and left SFA, whereas ICD was associated with the right BLA and left SFA. The left SFA plays an important role in sleepiness.

All four subregions exhibited overlapping and distinct hypo-connectivity profiles (Fig. 4b) correlated to emotional, olfactory, cognitive, sleep, and autonomic deficits. This means that the more severe the NMS in Fig. 4a, the lower the connectivity between the amygdala subregion and the brain region in Fig. 4b. Specifically, decreased connectivity between each subregion and the temporal lobe (including the bilateral TP and left MTG), and cingulate cortex commonly correlated to NMS. Distinct hypo-connectivity between different subregions and specific brain regions associated with NMS were also observed. The left CMA exhibited distinct hypo-connectivity with the left hippocampus, right MTG and SMA. The left BLA displayed a distinct hypo-connectivity from the right parahippocampus. The right BLA showed a distinct hypo-connectivity from the right OFC. The left SFA had distinct hypo-connectivity from the left OFC, putamen, and right hippocampus.

### Validation analysis

We validated our results by considering the following aspects. First, to avoid subregion selection bias due to separate PLS modeling of the four subregions, we used data from all four subregions to construct one PLS model to validate the association between FC changes in the amygdala and NMS (Supplementary Fig. 4). Second, we used one-way ANOVA tests on both raw data and harmonized data, with multi-sites as the factor, to examine whether our findings were influenced by specific sites. Significant main effects were observed on raw FC maps but not on harmonized FC maps (Supplementary Fig. 5 and Supplementary Table 7), indicating the site effect was corrected in the data. Third, to further examine whether the site effect had impact on our results, we removed the data from cohort 2 and performed FC analysis using only the data from cohort 1, and the validated results were consistent with the results of multicenter data (Supplementary Fig. 6 and Supplementary Fig. 7).

## Discussion

In this study, we investigated FC abnormalities in amygdala subregions in PD and further linked these subregional FC profiles to multiple NMS features of PD using multivariate PLS analysis. We obtained the following findings: (1) We identified common and distinct hypo-connectivity profiles of the amygdala subregions in PD. Overlapping hypo-connectivity with amygdala subregions such as OFC, vmPFC, temporal, insula cortex and striatum was observed mainly in the fear circuit. Distinct hypo-connectivity with the amygdala subregions was found mainly in the limbic circuit. (2) Common and distinct roles of amygdala subregional dysfunction to NMS were preliminary explored. Common roles of amygdala subregional dysfunction are manifested in emotional disturbances, hyposmia, cognitive deficits, pain, and sleep disorders. Distinct roles of amygdala subregional dysfunction to NMS are as follows. Left CMA, right BLA, and left SFA hypofunctions are involved in autonomic dysfunction, while bilateral BLA and left SFA hypofunctions are associated with fatigue. Right BLA and left SFA hypofunctions was associated with impulsivity, and left SFA hypofunction was associated with sleepiness.

### Spatial pattern of amygdala subregions’ FC alterations

Previous studies have examined the connectivity of the amygdala subregions in healthy adults. Bzdok^15^ and Roy^16^ et al. observed that the CMA, BLA, and SFA subregions shared common connectivity in the temporal and frontal regions. Unique connectivity was observed between the CMA and motor-related cortex, BLA and parietal cortex, and SFA and limbic lobe. Based on a relatively large multicenter sample, our results indicate that amygdala subregional FC in HCs is consistent with previous results (Fig. 1).

Group differences revealed that four subregions (left CMA, bilateral BLA, and left SFA) shared common hypo-connectivity with the fear and limbic cortico-striato-cortical circuits, including PFC, temporal lobe (TP, STG, parahippocampal gyrus), parietal cortex (SMG), insula, cingulate cortex, and putamen in PD (Fig. 2). These commonly hypo-connected areas are also collectively referred to as the default mode network (DMN) regions^20^. Decreased activities in DMN regions have been reportedly associated with depression^21^, cognitive deficits^22^, sleep^23^, and pain^24^. Additionally, the insula is involved in socio-emotional, sensorimotor, olfactory, and cognitive networks^25^, and amygdala-insula hypo-connectivity has been implicated in the pathophysiology of neuropsychiatric symptoms^26^, sleep disorders^27^, and fatigue^28^. The amygdala, insula, and basal ganglia are key regions that control and influence the autonomic nervous system, and hypo-connectivity between these areas is associated with autonomic dysfunctions^29^.

Despite the common hypo-connectivity, there is also differentiated hypo-connectivity between amygdala subregions in PD. All three amygdala subregions in the left hemisphere were significantly hypo-connected to the right amygdala, which impaired the effective coordination and integration of functional brain circuits within and between the hemispheres. Additionally, the left CMA is hypo-connected to the right SMA, postcentral gyrus, and left hippocampus, which are sensorimotor cortices. Hypo-connectivity of the amygdala with the sensorimotor cortices may result in motor and sensory processing dysfunction. The left CMA is also hypo-connected to the ACC, MCC, Nac, and insula, which are contained in the salience network (SN) regions^30^, and hypo-connectivity of the amygdala to the SN regions implicates disturbances in reward, emotion, and cognitive processing circuits. In summary, the left CMA is hypo-connected to the sensorimotor cortices and SN regions. The left and right BLA share similar hypo-connectivity patterns; both subregions are hypo-connected to the left ACC, vmPFC, bilateral OFC, and hippocampus, contained mainly in the fear circuit. Disrupted connectivity between the amygdala and fear circuit was observed in emotional dysregulation^31^. The bilateral BLA was also hypo-connected to the right olfactory cortex. Olfactory signals are transmitted directly from the olfactory bulb to the amygdala^32^. Therefore, a hypo-functioning amygdala may lead to impaired olfactory circuits. In summary, the bilateral BLA is hypo-connected to the fear and olfactory circuit. Finally, the left SFA is hypo-connected to the left olfactory cortex, as well as hypo-connected to the left Nac, MCC, vmPFC, pre- and postcentral gyrus, bilateral IFG, and OFC, which are sensorimotor cortices and are also involved in olfactory, reward, and emotion circuits. In summary, the left SFA is hypo-connected with the sensorimotor cortices, reward, and olfactory circuit. Moreover, a recent autopsy study based on a large sample of patients with Lewy body disease demonstrated that many patients followed an amygdala-centered distribution of Lewy pathology, whereby α-synuclein is deposited in the amygdala, temporal cortex, limbic system, and olfactory bulb^33^. These findings provide pathological evidence for disrupted connectivity of the amygdala in this study.

An interesting phenomenon in our study was the lateralization of amygdala functional impairment. All three subregions on the left side were impaired, whereas only the BLA on the right side was affected. Of all the patients with PD, 58.3% (67/115) exhibited right-side dominant motor symptoms. Therefore, damage to the left hemisphere is more pronounced in patients with PD. Symptom laterality is one of the main features of PD^34^, and pathological findings also support the concept that α-synuclein pathology initially propagates mainly in the ipsilateral hemisphere in most patients, leading to asymmetric dopaminergic dysfunction and parkinsonism^33^. It has been suggested that patients with PD with right-dominant motor deficits (corresponding to left hemisphere brain damage) develop more severe NMS^35^.

### Common and distinct roles of amygdala subregional hypo-connectivity in non-motor symptoms

PLS analyses revealed that age, brain atrophy, motor symptoms, and NMS, all factors that exacerbate the severity of PD^36^, are strongly associated with amygdala dysfunction. This is consistent with previous studies demonstrating that amygdala function is associated with age, brain morphology, motor symptoms, and NMS in PD^36,37^. Moreover, our study revealed decreased connectivity of all four amygdala subregions commonly associated with emotional disorders (depression, anxiety, and apathy), hyposmia, cognitive deficits, RBD, pain and hallucinations. All these symptoms are associated with amygdala dysfunction, suggesting that worsening of one or more risk factors may accelerate amygdala-centered circuit dysfunction and further accelerate the progression of PD. Accordingly, one study found that depressed patients with PD had more progressive cognitive decline and motor severity than non-depressed patients with PD^38^. Furthermore, a series of NMS that appeared during the prodromal stage of PD, such as hyposmia, RBD, depression, anxiety, and apathy^3^, were found to be associated with amygdala dysfunction in this study, suggesting that amygdala hypofunction may be a prodromal biomarker for PD, which requires further investigation in prodromal cohorts.

We identified a common impairment in the connectivity of all four amygdala subregions with TP, the left MTG, and MCC, as demonstrated by the FC features of the PLS-derived amygdala subregions. In physiological states, TP is associated with higher cognitive processes, such as visual, naming, and memory processing, as well as social and emotional processing^39^. The involvement of TP has been demonstrated in neuropsychiatric disorders, such as ICD, anxiety, and depressive disorders^40–42^. TP is also a part of swallowing-related cortices, and its hypo-connectivity is involved in dysphagia in PD^43^. Previous FC analyses identified MTG involvement in language, cognition, emotion, and auditory processing^44^. Impairments in MTG have been widely reported in cognition, depression, and ICD^45^. Furthermore, MCC has been reported to regulate motor, cognition, and emotion^46^. Overall, these are consistent with our findings that impaired amygdala connectivity with the TP, MTG and MCC is associated with anxiety, depression, apathy, and cognition.

Moreover, PLS analysis revealed different FC patterns among amygdala subregions. Hypo-connectivity in the left CMA with the right striatum, amygdala, MTG, left Nac, hippocampus, and IFG has been demonstrated. The striatum has the highest density of cholinergic neurons, while the amygdala, temporal cortex, Nac, and hippocampus receive dense cholinergic projections^47^. Molecular imaging studies revealed that these brain regions exhibit severe cholinergic deficits in PD, and cholinergic deficits have been shown to be associated with olfactory, cognitive, autonomic dysfunction (constipation), RBD, hallucination, and neuropsychiatric symptoms^47^. Meanwhile, these nodes with cholinergic deficits also spatially overlap with neural circuits involved in cognition, sleep, motor, visual, and autonomic functions^47^. Therefore, reduced connectivity of the left CMA to these cholinergic nodes may lead to a range of symptoms. The hypo-connectivity patterns in the left and right BLA are spatially similar. The bilateral BLA shares hypo-connectivity with the sensorimotor cortices (left parietal lobe and SMA, right striatum), as well as the left IFG and bilateral insula. Decreased activity in sensorimotor cortices have been reported in PD, which impairs sensory-motor processing and integration^48^ and is associated with hallucinations and difficulty swallowing. The IFG is connected with the OFC, vmPFC, and ACC and is involved in emotion, decision making^49^, and hypo-connection between the BLA and IFG, which may be associated with neuropsychiatric symptoms and ICD. Furthermore, a macaque study determined the extensive structural connections of the insula to the frontal, olfactory, parietal, temporal, somatosensory, and cingulate cortices using tracers^50^. The insular cortex is involved in pain, olfaction, emotion, decision making, fatigue, and autonomic function;^51^ thus, hypo-connection between the BLA and insula may be involved in these symptoms. Previous studies reported that insular pathology is associated with OH^52^, and we found that bilateral BLA are hypo-connected with the insula, which may be the mechanism by which OH is associated with the BLA. The right BLA also exhibits hypo-connection with the vmPFC and MCC; such hypo-connectivity has been reported in reward and pain processing^53,54^. Finally, the fear circuit such as the hippocampus, parahippocampus, Nac, and OFC show widespread hypo-connectivity with the left SFA, and amygdala-centered fear circuit dysfunction is involved in emotional symptoms and ICD^54^. Decreased FC of the frontal and limbic lobes is associated with sleepiness, and the decreased FC between the left SFA and the frontal and limbic lobes found in this study may be the mechanism underlying sleepiness^55^. A positron emission tomography study showed that urinary dysfunction is associated with altered function of the SMA, insula, and putamen^56^. We found that all subregions are hypo-connected to these brain regions, which may be the mechanism of amygdala involvement in urinary dysfunction. Emerging evidence suggests that the extended amygdala, including the bed nucleus of the stria terminalis (BNST), which modulates fear, anxiety, and reward^57^, is also a critical hub in regulating NMS. Future explorations of changes in BNST neurocircuitry have the potential to reveal new mechanisms underlying NMS of PD^58^.

Our study highlights a latent clinical-functional relationship among amygdala subregions in PD. However, several important limitations should be considered when interpreting these findings. First, our results were based on cross-sectional data and excluded extrapolation to longitudinal progression. Future studies should validate these amygdala dysfunctional patterns and their contributions in larger cohorts of patients and explore whether the brain-behavior signature identified in our study is consistent across disease stages. Second, some clinical rating scales for NMS used in this study may not be the most sensitive scale. For example, for anxiety, Parkinson Anxiety Scale^59^ is more sensitive than STAI. The MDS-UPDRS Part I items are also not the preferred scale for evaluating NMS. Moreover, in this study, 84.3% (97/115) patients with PD showed impaired olfaction, 46.1% (53/115) patients showed global cognitive deficits, 47.0% (54/115) patients with RBD, 28.7% (33/115) patients with anxiety, 22.6% (26/115) patients with depression, and 9.6% (11/115) patients showed ICD. Thus, some NMS included in this study were at subclinical stages (e.g., QUIP, GDS, and STAI scores in most patients were below the cut-off for clinical diagnosis), which have the potential to weaken the brain-behavioral associations of NMS in PD. Future studies should use more accurate scales and include more patients above the symptomatic cut-offs to uncover more accurate brain-behavior relationships. Third, this study used the ComBat method to harmonize gradient measures across sites. However, potential nonlinearities and interaction effects between groups may require better methods (nonlinear models) to identify and correct for multicentric effects. Finally, despite the discussion of resting-state functional networks (RSFN) such as DMNs, the present study did not investigate the connectivity within and between different RSFN. Future studies could specifically examine the connections between amygdala subregions and individual RSFN.

In conclusion, we observed significant lower functional connectivity of the amygdala subregions in PD. In addition, the preliminary brain-behavior relationship identified in this study suggests that amygdala subregions have common, as well as distinct roles in NMS in PD. Overall, our study provides new insights into the neurobiological mechanisms of NMS associated with amygdala dysfunction in PD.

## Methods

### Participants

A total of 248 participants were enrolled in this study. Twenty patients with PD and 14 HCs were excluded due to excessive head movements; 21 HCs were excluded due to cognitive impairment, anxiety, or sleep disorders. Therefore, we enrolled 193 participants (PD: 115, HC: 78) from two independent cohorts. Specifically, 157 participants (PD: 86, HC: 71) from cohort 1 were recruited from June 2017 to May 2021 from the Department of Neurology, Xuanwu Hospital Capital Medical University. In cohort 2, 36 participants (PD: 29, HC: 7) were obtained from the Parkinson’s Progression Markers Initiative (PPMI) database (https://www.ppmi-info.org/). Notably, to reduce the effects of dopamine drugs on brain activity, we only included patients who were in the “OFF medication” state (at least 12 h dopaminergic drug withdrawal) during MRI scans in both cohorts. This study was approved by the Ethics Committee of Xuanwu Hospital of Capital Medical University. All participants provided written informed consent before inclusion. Patients with PD were diagnosed according to the Movement Disorders Society (MDS) Clinical Diagnostic Criteria^60^. The HCs were recruited from the community. The exclusion criteria were as follows: (1) having an age below 40 or above 80, (2) patients with genetic/familial PD, (3) the presence of confounding neurological diseases, (4) HCs with motor impairment, or cognitive/behavioral abnormalities, and (5) having any MRI contraindications, or excessive head movement during scanning. In addition, as the lateralization of brain structure and function is suggested to be associated with handedness, to avoid irrelevant variance in brain structures and functions across subjects and to raise statistical sensitivity, left-handed participants were excluded in this study.

### Clinical and behavioral assessments

All participants underwent clinical assessment of motor symptoms and NMS in the “OFF medication” state. Motor symptoms were evaluated using the MDS-UPDRS-III. The dosage of antiparkinsonian drugs was calculated using the LEDD. Olfactory function was assessed using the University of Pennsylvania Smell Identification Test (UPSIT). Cognitive ability was evaluated using the Montreal Cognitive Assessment (MOCA). The presence of depression, anxiety, apathy, hallucinations, pain, urinary disorder, constipation, orthostatic hypotension, fatigue, drooling, and swallowing was measured using the Geriatric Depression Scale (GDS), State-Trait Anxiety Inventory (STAI), and MDS-UPDRS I-1.5, I-1.2, I-1.9, I-1.10, I-1.11, I-1.12, I-1.13, II-2.2, and II-2.3, respectively^61^. Trait impulsivity was estimated using a questionnaire for impulsive-compulsive disorder (QUIP)–current short^62^. A positive ICD symptom was scored as 1 and a negative ICD symptom was scored as 0. Sleep disturbances were measured using the Rapid Eye Movement Sleep Behavior Disorder Screening Questionnaire (RBDSQ) and Epworth Sleepiness Scale (ESS) (Table 1).

### MRI acquisition and preprocessing

During MRI scanning, participants were instructed to keep eyes closed, stay awake, and not think of anything particular. All the patients were scanned in the “OFF medication” state. The MRI data in cohort 1 were acquired using a Siemens Magnetom Skyra 3 T scanner (Erlangen, Germany). Rs-fMRI images were obtained using a single-shot spin-echo echo-planar imaging (SE-EPI) sequence. Rs-fMRI images were obtained with the following parameters: repetition time (TR), 2000 ms; echo time (TE), 30 ms; field of view (FOV), 22 × 22 cm^2^; flip angle, 90°; voxel size, 3.4 × 3.4 × 3 mm^2^; 35 slices, no gap; 176 repetitions; scanning time, 5 min 52 s. T1-weighted anatomic images were scanned using a magnetization-prepared 3D rapid gradient echo (MPRAGE) sequence. The image parameters were as follows: TR/TE/flip angle, 2530 ms/2.98 ms/7°; FOV, 25.6 × 25.6 cm^2^; voxel size 1 × 1 × 1 mm^2^; 192 slices, no gap. The acquisition time was 5 min 13 s. MRI data from the PPMI dataset were scanned with Siemens Trio 3 T scanners, using the same acquisition protocol to ensure data standardization. In brief, rs-fMRI images were obtained using the SE-EPI sequence. The imaging parameters were as follows: TR/TE/flip angle, 2400 ms/25 ms/80°; FOV, 22 × 22 cm^2^; voxel size, 3.3 × 3.3 × 3.3 mm^2^; 40 slices, no gap. 210 repetitions; scanning time, 8 min 24 s. T1-weighted anatomical images were scanned using an MPRAGE sequence. The imaging parameters were as follows: TR/TE/flip angle, 2300 ms/3 ms/9°; FOV, 25.6 × 24 cm^2^; voxel size, 1 × 1 × 1 mm^2^; 170 slices, no gap. Imaging data were preprocessed and analyzed using DPABI (v6.0; http://www.rfmri.org/dpabi), including removal of the first ten volumes, slice timing correction, head motion correction, tissue segmentation, spatial normalization into Montreal Neurological Institute (MNI) space and resampling into 3-mm isotropic voxels, spatial smoothing with a full width at half maximum of 6 mm Gaussian kernel, and temporal bandpass filtering at 0.01–0.08 Hz. Notably, participants were excluded if their mean framewise displacement (FD) was >0.3 mm, or if the translational/rotational movements were >3 mm or 3°^18^.

### Functional connectivity analysis of amygdala subregions

**Fig. 5:**
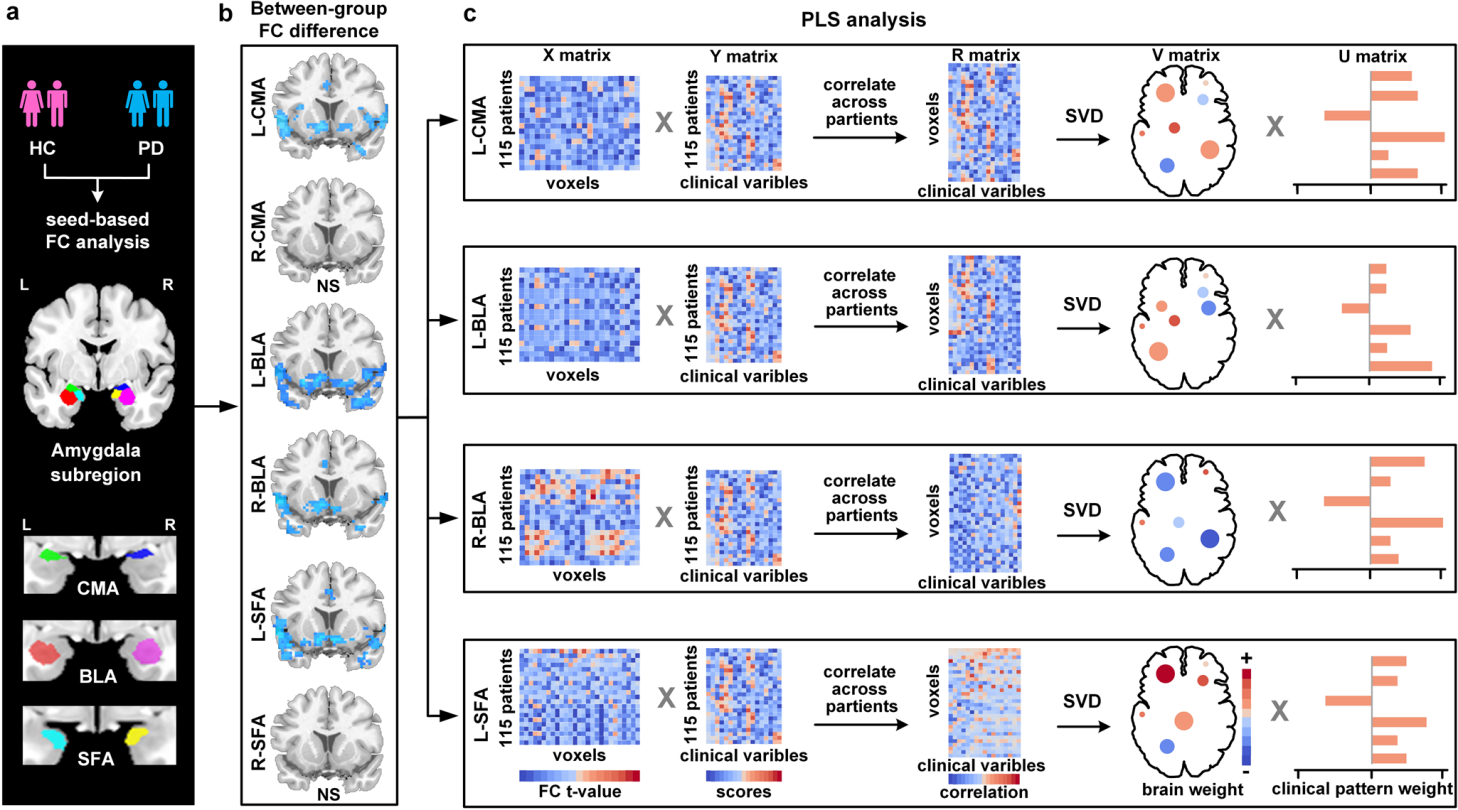
Partial least-squares (PLS) analysis framework. **a** Amygdala subregional seed based functional connectivity (FC) analysis is performed separately. **b** Four subregions (left CMA, left BLA, left SFA, and right BLA) present statistical differences between the two groups. NS no significance. **c** Group-difference FC maps are used to construct subregion-specific brain matrices. PD-related clinical features are used to construct behavior matrices. Subregion-specific brain and behavior matrices are subsequently correlated across patients respectively. Singular value decomposition (SVD) is applied and subregion-specific brain weights, behavior weights are output, respectively.

Amygdala subregional templates (CMA, BLA, and SFA) were generated using the JuBrain Anatomy Toolbox^63^ (https://www.fz-juelich.de/inm/inm-7/EN/Resources/_doc/SPM%20Anatomy%20Toolbox_node.html). Given the lateralization patterns of amygdala subregional functions and connections^64^, masks for each subregion were created separately for each hemisphere. Voxels were classified as potential amygdala subregions only if the probability of their assignment to the amygdala subregion was higher than that of other adjacent structures (>10% probability). Each voxel was assigned to only one subregion, resulting in nonoverlapping amygdala subregions. We analyzed FC using DPABI software. For each amygdala subregion, a whole-brain FC map was obtained for each participant by calculating Pearson’s correlation coefficients between the mean time series of all voxels in the subregion and the time series of every voxel within the GM. The correlation coefficients were converted to z-scores using Fisher’s r–z transformation to improve the normality. Six z-scored FC maps were generated for each participant (Fig. 5a). Finally, we applied ComBat Harmonization (https://github.com/Jfortin1/ComBatHarmonization) to all subjects to correct for scanner/site effects on the FC maps.

### Grey matter volume analysis of amygdala subregions

Given that associations between changes in GM volume and NMS in PD were previously reported^65,66^, we used FSL tool (v5.0, http://fsl.fmrib.ox.ac.uk/) to perform tissue segmentation and volume estimation. The TIV, whole brain GM, WM, and CSF volumes were first calculated using FSL-FAST. For the amygdala, FSL-FIRST was used to automatically detect the position of the amygdala based on shape and intensity, based on a Bayesian probabilistic model. Then, a pre-defined and learned model was applied to search through the best combinations of shape mode and obtain the most probable shape instance^67^. Finally, the labels of amygdala for each subject in T1-weighted native space were obtained. We next performed nonlinear registrations to align individual T1-weighted images and MNI152 T1-weighted image using ANTs (v2.0, https://github.com/ANTsX/ANTs), and applied the inverse transformation to the amygdala subregional atlas in the MNI152 space, resulting in the subject-specific subregional atlas in the structural native space. Together, Labels of six amygdala subregions were generated as masks, and the volume of amygdala and amygdala subregions were assessed.

### Statistical analysis

#### Between-group differences of demographics, clinical features, and FC maps

Statistical analyses for demographic and clinical features were performed using SPSS 22.0 (IBM, NY, USA). The Kolmogorov-Smirnov (K-S) test was used to test whether the data obeyed a normal distribution. Two-sample t tests (for age, UPDRS III, GM, TIV, and amygdala subregional volumes) and Mann-Whitney U tests (for education, motor symptoms except UPDRS III, and NMS) were used to determine differences between the two groups (chi-square test for gender differences). GM and amygdala volumes were normalized using the TIV. SPM12 (https://www.fil.ion.ucl.ac.uk/spm/software/spm12/) was used to calculate within- and between-groups differences in FC. BrainNet Viewer (v1.7, https://www.nitrc.org/projects/bnv/) was used for results visualization. For the FC maps of each amygdala subregion, we first applied one-sample *t*-tests to the PD and HC groups to determine the connectivity profile of each subregion. The significant level for the within-group test was set at a family-wise error (FWE) corrected *P* < 0.05 at voxel-level with a cluster size ≥10 voxels. An FC mask was then generated as the union of the within-group results from both groups for each subregion. Between-group differences in FC maps for each amygdala subregion were then measured using the general linear model within this mask, with age, sex, and education as covariates, and the significant level was FDR corrected *P* < 0.05 at the voxel level with a cluster size ≥10 voxels.

### Multivariate associations between FC maps and clinical features

We performed PLS correlation analysis to examine the relationship between amygdala subregional FC and clinical features using the myPLS package (https://github.com/danizoeller/myPLS). PLS analysis combines clinical information and brain function and allows the identification of covariate patterns, that is, a set of brain patterns that significantly correlate with a set of clinical patterns^68,69^. In the present analysis, we included only subregions that exhibited significant differences in FC between the PD and HC groups. Therefore, only four subregions (the left CMA, left BLA, left SFA, and right BLA) presented statistically significant FC differences between the two groups and were used to construct the brain matrix. In addition, several demographic (age, gender) and clinical features (motor severity, dopamine drugs) have been reported to affect the severity of NMS^70,71^ and the function of the amygdala subregions^54^. As such, motor symptoms, medication, GM and amygdala volumes, and head movement values were regressed from the imaging and NMS data using multiple linear regression to eliminate the confounding effects of these variables. Moreover, we constructed separate PLS models for each subregion to facilitate the identification of the common and distinct contributions of the subregions on the NMS.

First, the PLS model’s inputs were imaging matrix **X** (**X** = **N** × **M**; **N** is the number of included patients and **M** is brain voxels) and behavioral matrix **Y** (**Y** = **N** × **B**; **B** is the behavior score) (Fig. 5b). Subsequently, **X** and **Y** were z-scored, and the correlation matrix **R** (i.e., **R** = **X**^*T*^ **Y**) was constructed separately. Singular value decomposition (SVD) was then applied to the resulting **R** matrices, yielding three matrices from each **R** (**R** = **X**^*T*^ **Y** = **SUV**^*T*^)^72^. The PLS model searches for latent variables (LVs) that express the maximum amount of common information about **X** and **Y**. These LVs have the maximum covariance, which is reflected in the singular values that constitute matrix **S** (**S** = **L** × **L, L** is LV)^68^. The first LV consists of the 1st column vector of **U** (**U** = **B** × **L**), the 1st column vector of **V** (**V** = **M** × **L**), and the 1st column vector of the singular values matrix **S**. The number of LVs was equal to the number of columns of **Y**. **U** is the left singular vector of **R** that represents behavioral weights that best characterize **R**. **V** is the right singular vector of **R** representing voxel weights that best characterize **R**. Finally, brain scores (**L**_X_ = **XV**) and behavior scores (**L**_Y_ = **YU**) were calculated to characterize how strongly an individual contributes to the LV pattern^73^.

Statistical significance of each LV was determined using permutation tests. Specifically, we held the matrix **Y** constant and randomly rearranged the patients’ order in matrix **X** 1000 times. Hence, we effectively removed the dependencies between the brain and behavior. We then reran the SVD for the permuted data and obtained a null distribution of the singular values. *P*-values were used to estimate the probability that the permuted singular value exceeded the observed singular value. LVs with *P* values < 0.05 were considered significant.

Subsequently, for each significant LV, bootstrap resampling was used to assess the reliability of the brain and behavioral weights. Specifically, we randomly resampled the subjects’ order in both matrices **X** and **Y** 1000 times and performed SVD to obtain the distribution of bootstrap-estimated **S**, **U**, and **V**. The reliability of the observed weights (i.e., **U** and **V**) was calculated using the BSR, that is, the ratio of each weight to the standard error of bootstrap-estimate weights. The larger the BSR of the voxel, the more it contributes to the LV and the greater its stability across patients. BSR is equivalent to z-scores and a BSR threshold of ±3.3 is usually considered to be *P* < 0.001. Moreover, a behavioral variable was regarded as significant if the 95% confidence interval (CI) of the bootstrapping distributions did not cross zero.

### Reporting summary

Further information on research design is available in the Nature Research Reporting Summary linked to this article.

## Supplementary information

Supplemental materials

Reporting Summary

## Data Availability

The PPMI dataset (https://www.ppmi-info.org/) used for this study is available for qualified researchers through application. The Xuanwu Hospital dataset used in this study is available for qualified researchers to request by contacting the corresponding author.

## Code availability

SPM12 (https://www.fil.ion.ucl.ac.uk/spm/software/spm12/), DPABI (v6.0, http://www.rfmri.org/dpabi), FSL (v5.0, http://fsl.fmrib.ox.ac.uk/), Combat (https://github.com/Jfortin1/ComBatHarmonization), and PLS (https://github.com/danizoeller/myPLS) used in the study are open sources.
