# Author Correction: Common and distinct roles of amygdala subregional functional connectivity in non-motor symptoms of Parkinson’s disease

**DOI:** 10.1038/s41531-023-00506-z

**Published:** 2023-04-10

**Authors:** Junling Wang, Lianglong Sun, Lili Chen, Junyan Sun, Yapei Xie, Dezheng Tian, Linlin Gao, Dongling Zhang, Mingrui Xia, Tao Wu

**Affiliations:** 1 Center for Movement Disorders, Department of Neurology, Beijing Tiantan Hospital, Capital Medical University, Beijing, 100070 China; 2 State Key Laboratory of Cognitive Neuroscience and Learning, Beijing Normal University, Beijing, 100091 China; 3 Beijing Key Laboratory of Brain Imaging and Connectomics, Beijing Normal University, Beijing, 100091 China; 4 IDG/McGovern Institute for Brain Research, Beijing Normal University, Beijing, 100091 China; 5 Department of General Medicine, Tianjin Union Medical Center, Tianjin, 300122 China

**Keywords:** Parkinson's disease, Parkinson's disease

Correction to: *npj Parkinson’s Disease* 10.1038/s41531-023-00469-1, published online 17 February 2023

“The online version is incorrectly labeled that Junling Wang, Lianglong Sun, Mingrui Xia & Tao Wu are the co-first authors. Mingrui Xia & Tao Wu are not co-first authors, but co-correspondents. The original article is now corrected”.

