# Supplementary material for: Common and distinct roles of amygdala subregional functional connectivity in non-motor symptoms of Parkinson’s disease
Source: NPJ Parkinsons Dis. 2023 Feb 17;9:28. doi: 10.1038/s41531-023-00469-1 (PMC9938150; doi:10.1038/s41531-023-00469-1)
Supplement: Supplementary file 1 — Supplemental materials [file 41531_2023_469_MOESM1_ESM.pdf]

Supplementary Materials

Supplementary Figures

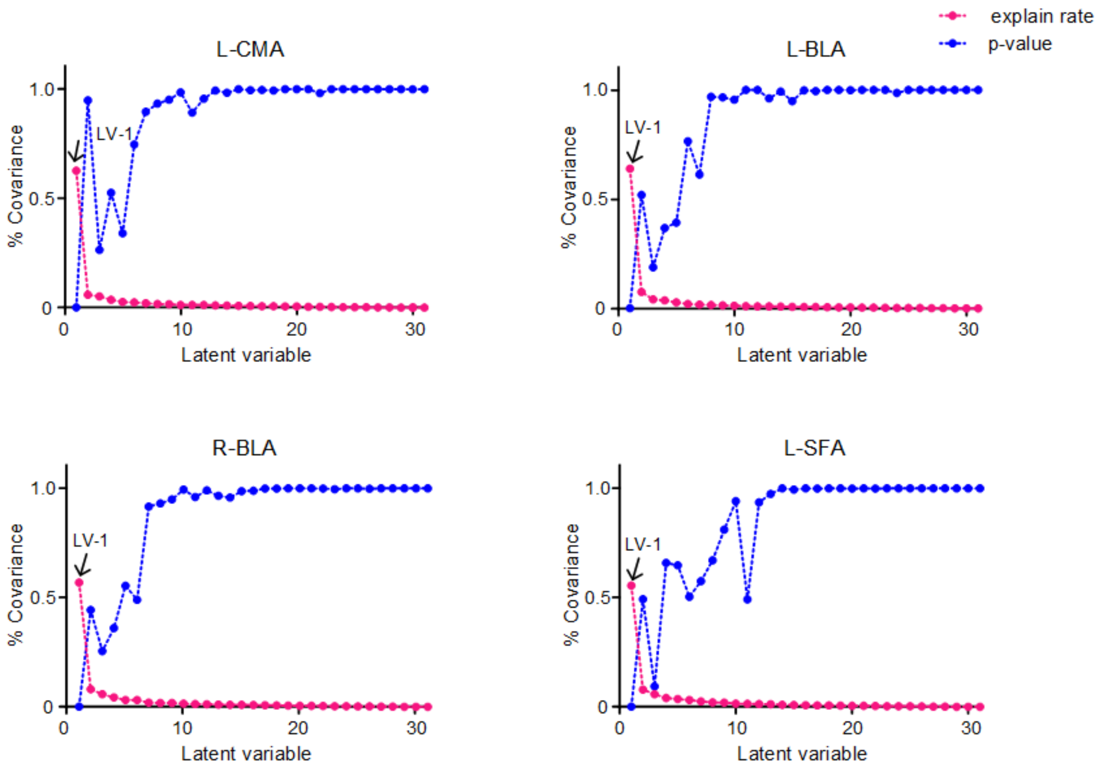

Supplementary Figure 1. Covariance explained rates and permutated P values for all latent variables (LVs) in each amygdala subregion in the in PLS analysis.

Demographics, medication, amygdala volume, motor symptoms, and non-motor symptoms (NMS) were included into the PLS model. Each subregion identified one statistically significant LV, respectively.

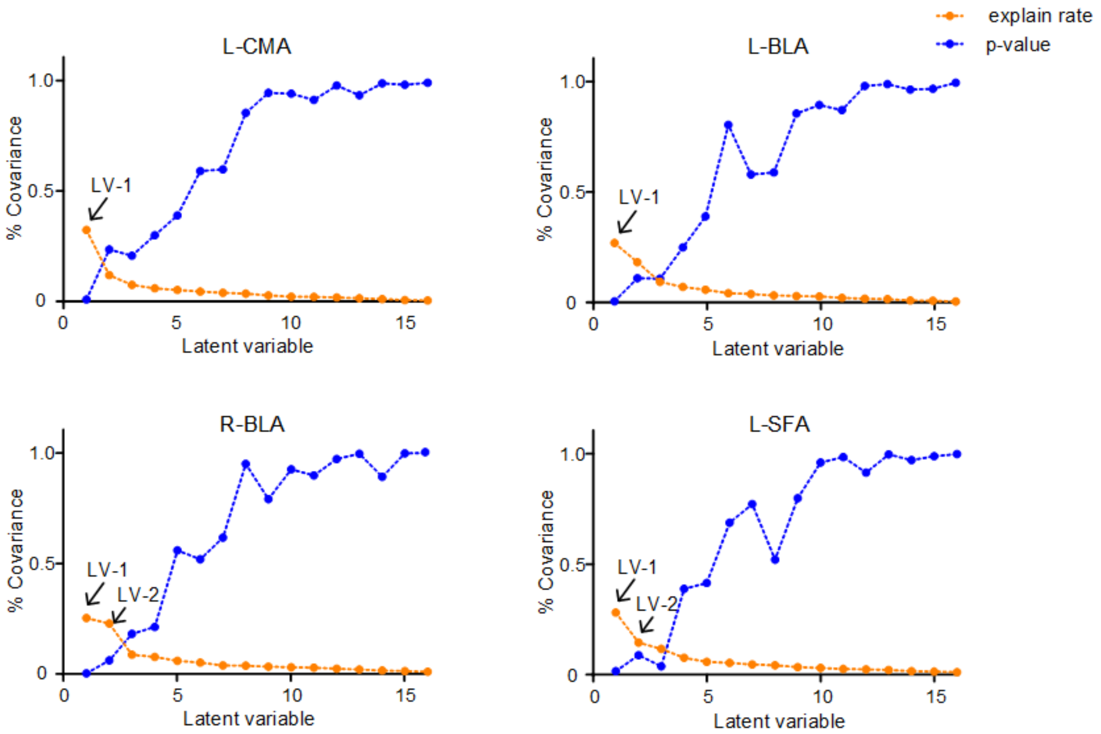

Supplementary Figure 2. Covariance explained rates and permutated P values for the latent variables (LVs) in each amygdala subregion in NMS-specific PLS analysis. Demographic characteristics (gender, age, and education), clinical features (disease duration, UPDRS III, and H-Y stage), medication (LEDD), and GM and amygdala volume were regressed in both NMS scores and FC maps and re-performed the NMS-specific PLS analysis. Left CMA and left BLA identified one statistically significant LV, respectively. Right BLA and left SFA identified two statistically significant LVs, respectively.

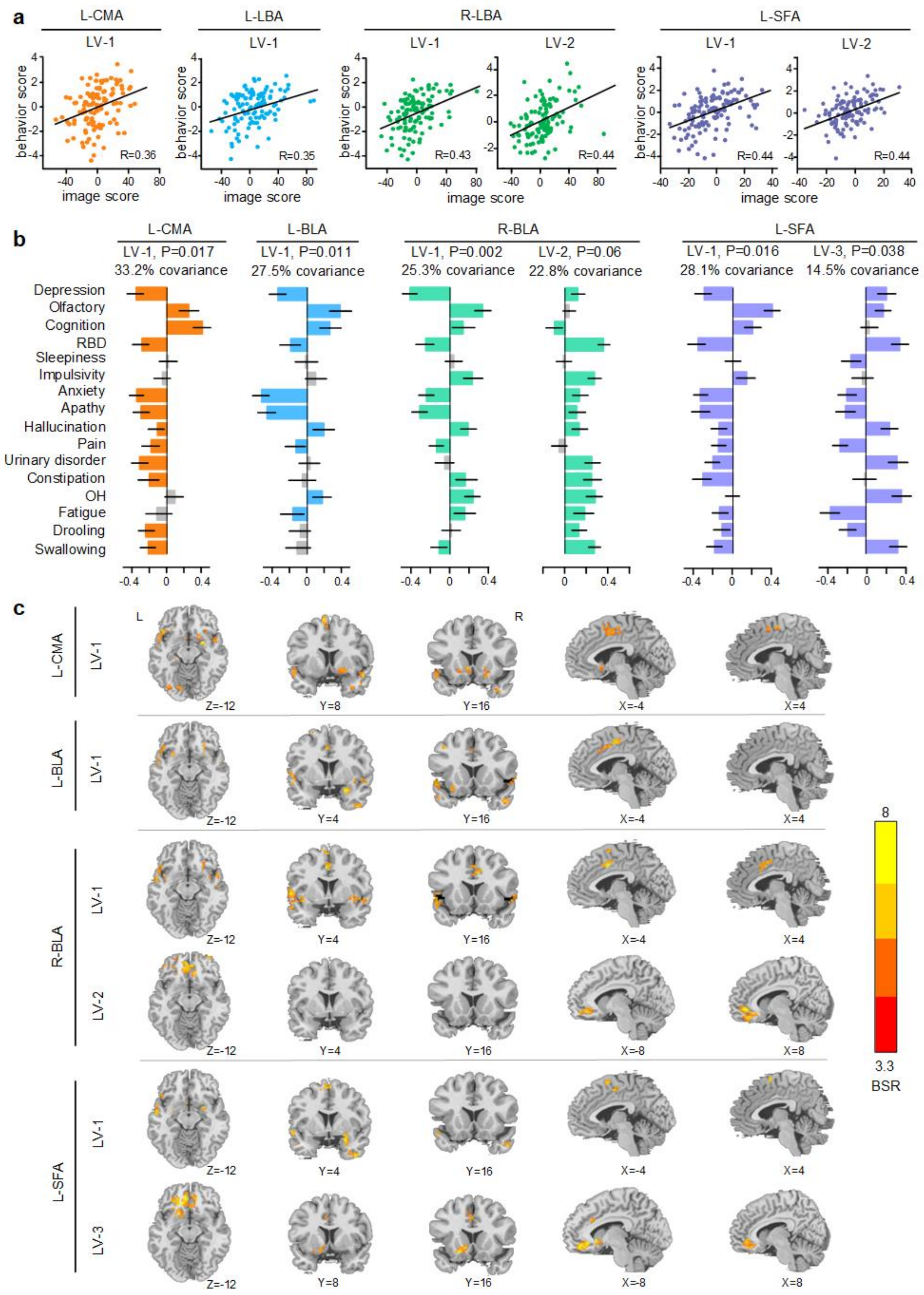

Supplementary Figure 3. NMS specific-functional signatures of the amygdala subregions in PD.

a Brain scores and behavior scores of LV-1 demonstrate significant correlations in each

subregion. Individuals who exhibited the FC pattern in (**c**) tend to exhibit corresponding behavioral phenotypes in (**b**). **b** Subregion-specific NMS features. The contribution of each subregion to each NMS is shown using correlation coefficients. Error bars indicate bootstrap estimated 95% confidence intervals of correlation strength. **c** Subregion-specific FC maps associate with NMS. Patients who exhibit these FC patterns tend to score worse regarding NMS severity. The contribution of dysconnectivity voxels on NMS is shown using bootstrap ratio (BSR), thresholding at BSR > 3.3 (P < 0.001, MNI 152 space). OH = orthostatic hypotension.

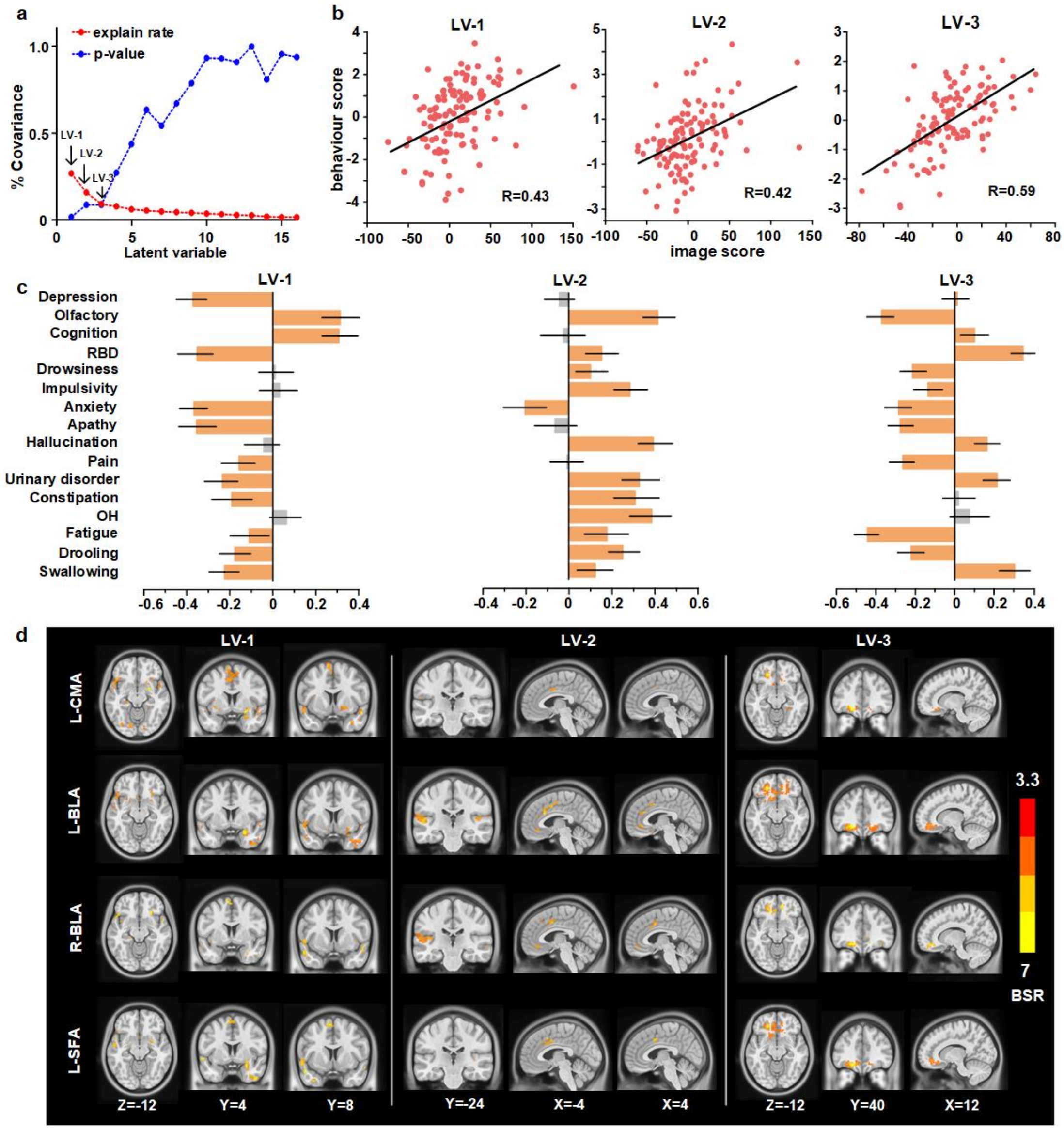

Supplementary Figure 4. Amygdala subregion-related NMS-functional signatures in the PLS model validation.

For model validation, four subregional data was combined to construct one PLS model instead of modeling each subregion separately. **a** Covariance explained rates and permutated P values for all latent variables (LVs). Validated PLS analysis identified 3 statistically significant LVs. **b** Brain scores and behavior scores of corresponding LVs exhibited significant correlations. **c** NMS features of LVs. The contribution of each NMS was shown using correlation coefficients. Error bars indicated bootstrap estimated 95%

confidence intervals of correlation strength. **d** Subregion-specific contributed FC maps associated with NMS. Patients who exhibit these FC patterns tend to score higher on NMS severity. The contribution of voxels was shown using BSR, thresholding at BSR > 3.3 (P<0.001, MNI152 space).

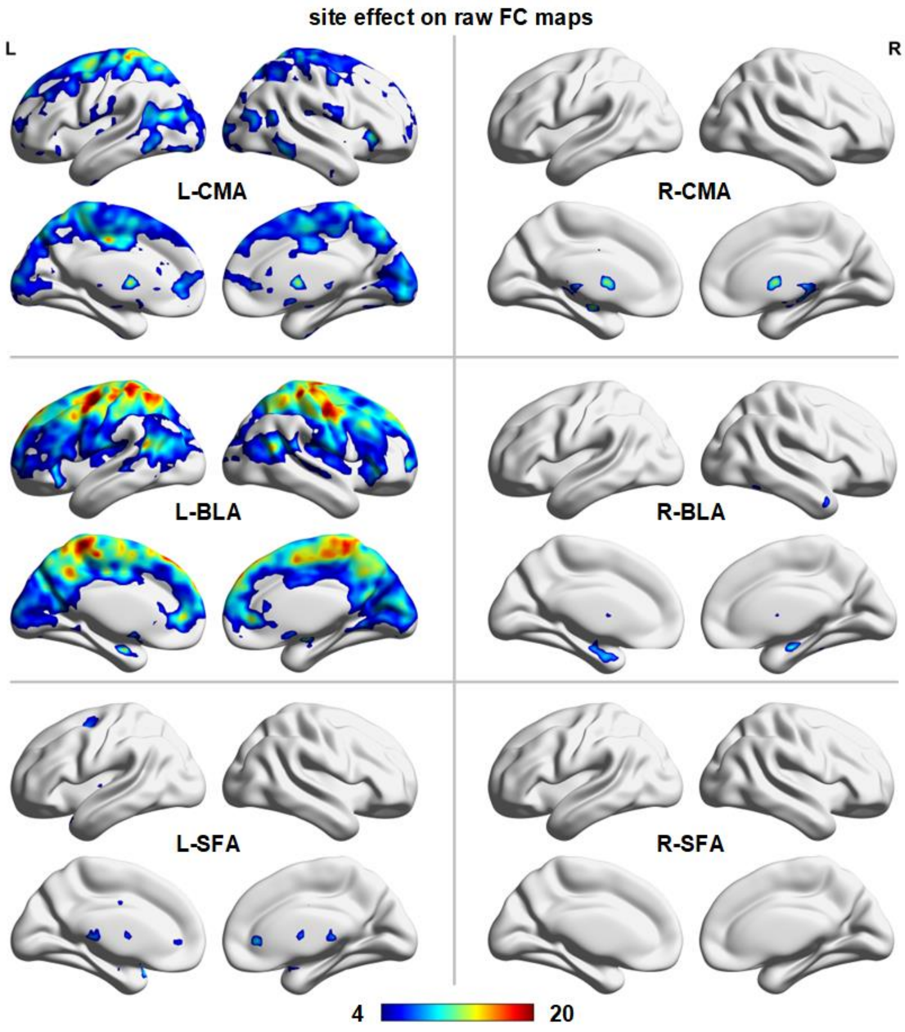

Supplementary Figure 5. Site effect on raw FC maps.

One-way ANOVA tests were performed on the functional connectivity of the raw data and harmonized data, with site as the factor. Significant main effects were observed in raw data in each amygdala subregions (FDR<0.05). The harmonized FC maps showed no

statistical significance.

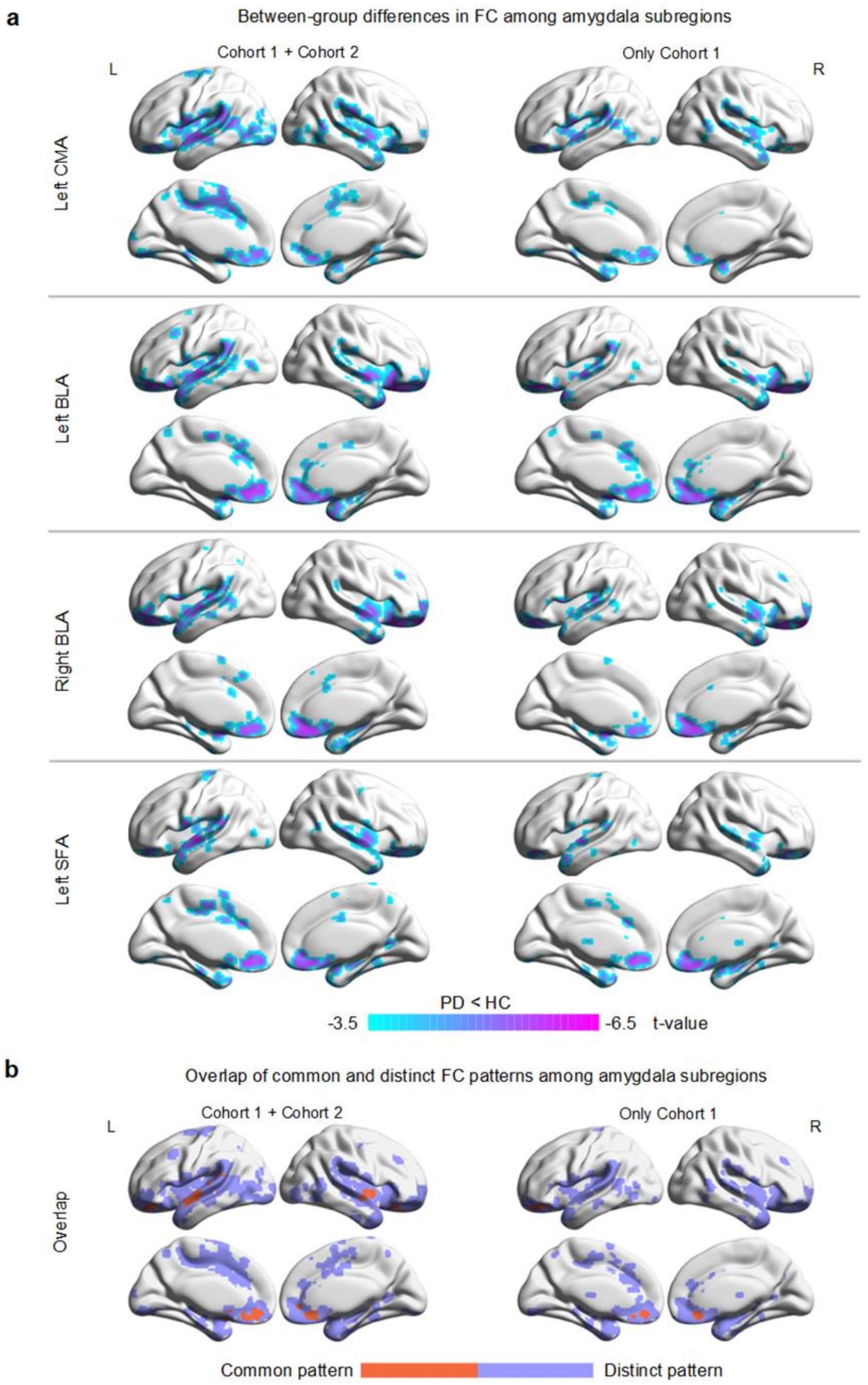

Supplementary Figure 6. Comparison of between-group differences in functional connectivity of single- (Cohort 1) and multicenter data (Cohort 1 + Cohort 2). Since the data from cohort 1 (PD: 86, HC: 71) were approximately three times larger than those from cohort 2 (PD: 29, HC: 7), and participant in cohort 2 was too small to be used as an independent validation dataset, so we removed all data from cohort 2 (removed the differences in imaging acquisition and center / scanner), used only the data from cohort 1, and re-performed the between-group FC changes to validate whether the statistical power is still reliable. The validation results showed that using data from cohort 1, we obtained amygdala dysconnectivity profiles similar to the results from the multicenter data. **a** Significant group differences in FC for each amygdala subregion. Group-difference FC maps are thresholded at FDR-corrected voxel-level $P < 0.05$ (MNI 152 space, cluster size ≥10 voxels). **b** Spatial similarities and differences of FC maps among amygdala subregions.

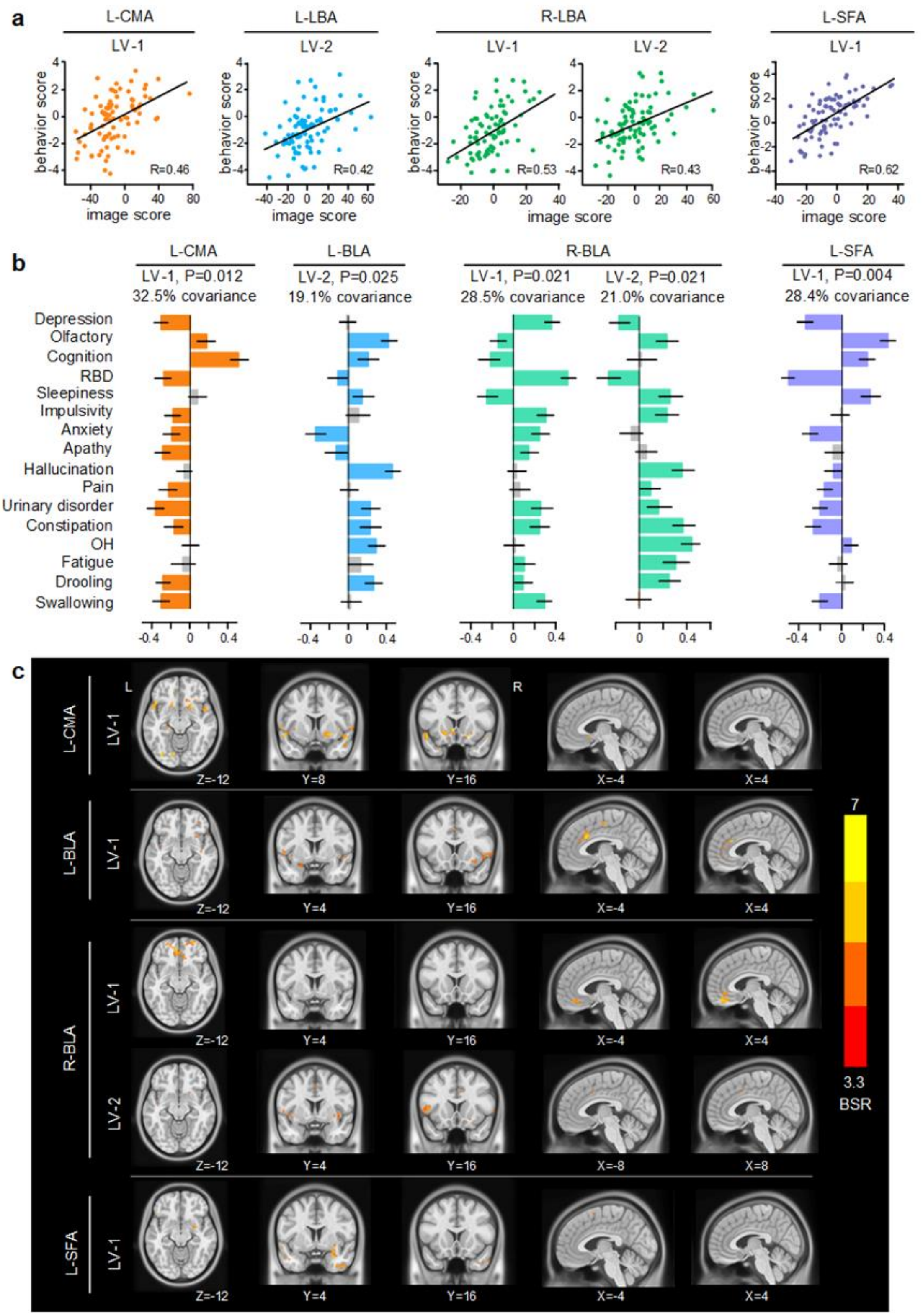

Supplementary Figure 7. NMS specific-functional signatures of the amygdala subregions in PD using data from cohort 1. The validation results showed that using data from cohort 1, we obtained brain-behavior associations similar to the results from the

multicenter data. **a** Brain scores and behavior scores of LV-1 demonstrate significant correlations in each subregion. Individuals who exhibited the FC pattern in (**c**) tend to exhibit corresponding behavioral phenotypes in (**b**). **b** Subregion-specific NMS features. The contribution of each subregion to each NMS is shown using correlation coefficients. Error bars indicate bootstrap estimated 95% confidence intervals of correlation strength. **c** Subregion-specific FC maps associate with NMS. Patients who exhibit these FC patterns tend to score worse regarding NMS severity. The contribution of dysconnectivity voxels on NMS is shown using bootstrap ratio (BSR), thresholding at BSR > 3.3 (P < 0.001, MNI 152 space). OH = orthostatic hypotension.

Supplementary Tables

Supplementary Table 1. Volume characteristics of included subjects.

**Morphological features, cm^3**	**PD group**	**HC group**	**P value**
Total intracranial volume	1862.15 ± 145.10	1823.67 ± 145.23	0.072
Grey matter volume [a]	791.76 ± 21.04	805.41 ± 21.50	**<0.001**
Left CMA volume [a]	0.40 ± 0.03	0.40 ± 0.04	0.565
Right CMA volume [a]	0.39 ± 0.03	0.39 ± 0.03	0.785
Left BLA volume [a]	1.51 ± 0.15	1.52 ± 0.15	0.603
Right BLA volume [a]	1.40 ± 0.13	1.40 ± 0.13	0.924
Left SFA volume [a]	0.55 ± 0.07	0.56 ± 0.06	0.612
Right SFA volume [a]	0.58 ± 0.07	0.58 ± 0.06	0.841

[a] Grey matter and amygdala volumes were normalized by regressing total intracranial volume. Values are presented as mean ± standard deviation (SD). Bold values indicate significant differences between two groups.

Supplementary Table 2. Connectivity differences between HC and PD participants for amygdala subregions.

Seed ROI, contrast		***Voxel-level***		***MNI [mm]***		
cluster size	***Hemisphere***	***Brain region***	***Peak t-value***	***x***	***y***	***z***
Left CMA, PD<HC						
3694	R	Superior Temporal gyrus	-5.54	51	-6	0
	L	Nucleus accumbens	-5.47	-9	12	-9
	L	Rolandic operculum	-5.43	-57	9	0
	L	Superior Temporal gyrus	-5.42	-51	-39	18
	L	Temporal pole	-5.35	-57	9	-6
	R	Insula	-5.19	51	-3	0
	L	Olfactory cortex	-5.17	-18	6	-15
	R	Temporal pole	-5.12	63	9	0
	R	Heschl's gyrus	-5.03	54	-6	3
	L	Heschl's gyrus	-4.96	-36	-24	6
	L	Insula	-4.91	-36	-18	3
	R	Rolandic operculum	-4.75	51	-3	3
	L	Putamen	-4.71	-12	9	-9
	R	SupraMarginal gyrus	-4.63	63	-21	21
	L	Inferior frontal gyrus	-4.63	-48	15	-9
	L	Gyrus rectus	-4.58	-12	15	-12
	L	ventromedial prefrontal cortex	-4.58	-9	45	-12
	R	Posterior orbital frontal cortex	-4.53	39	24	-21
	L	Anterior orbital frontal cortex	-4.50	-21	42	-15
	L	SupraMarginal gyrus	-4.48	-60	-24	15
	L	Middle temporal gyrus	-4.39	-57	-15	-6
	R	ventromedial prefrontal cortex	-4.34	12	36	-15
	R	Amygdala	-4.30	24	0	-21
	L	Posterior orbital frontal cortex	-4.30	-45	24	-15
	L	Inferior occipital gyrus	-4.28	-33	-93	-9
	R	Putamen	-4.12	18	9	-9
	R	Parahippocampal gyrus	-4.01	24	3	-24
	R	Inferior frontal gyrus	-3.97	33	36	-9
	L	Medial orbital frontal cortex	-3.93	-18	45	-21
	R	Postcentral gyrus	-3.84	63	-3	15
	R	Nucleus accumbens	-3.75	15	12	-9
	R	Gyrus rectus	-3.71	12	27	-15
	R	Medial orbital frontal cortex	-3.47	21	15	-21
76	L	Postcentral gyrus	-5.06	-57	-18	15
	L	Precentral gyrus	-3.86	-27	-24	72
585	L	Middle cingulate gyrus	-4.82	-3	6	42
	L	Supplementary motor area	-4.38	-3	6	45
	R	Middle cingulate gyrus	-4.02	3	6	39

	R	Supplementary motor area	-3.62	12	6	60
	L	Anterior cingulate gyrus	-3.67	-6	27	30
125	R	Inferior temporal gyrus	-4.58	51	-54	-6
	R	Middle temporal gyrus	-4.46	51	-54	-3
	R	Fusiform gyrus	-3.81	39	-9	-36
59	L	Lingual gyrus	-4.40	-21	-72	-6
35	R	Inferior occipital gyrus	-4.14	45	-84	-9
29	L	Hippocampus	-3.93	-24	-33	-9
	L	Parahippocampal gyrus	-3.72	-21	-33	-12
118	L	Middle occipital gyrus	-3.85	-45	-69	-3
15	R	Lingual gyrus	-3.60	15	-78	-12

Seed ROI, contrast		***Voxel-level***		***MNI [mm]***		
cluster size	***Hemisphere***	***Brain region***	***Peak t-value***	***x***	***y***	***z***
Left BLA, PD<HC						
3816	R	Posterior orbital frontal cortex	-5.92	27	21	-21
	R	Insula	-5.89	27	21	-18
	L	Anterior orbital frontal cortex	-5.77	-21	48	-15
	L	ventromedial prefrontal cortex	-5.65	-9	45	-12
	L	Middle temporal gyrus	-5.55	-57	-15	-3
	R	Medial orbital frontal cortex	-5.42	18	30	-21
	L	Olfactory cortex	-5.36	-15	9	-18
	L	Gyrus rectus	-5.32	-12	15	-12
	L	Nucleus accumbens	-5.31	-9	15	-9
	L	Superior temporal gyrus	-5.30	-57	-12	-3
	R	Gyrus rectus	-5.16	6	21	-21
	L	Anterior cingulate gyrus	-5.08	-6	36	-9
	R	ventromedial prefrontal cortex	-4.98	3	27	-15
	L	Temporal pole	-4.85	-27	6	-21
	R	Inferior frontal gyrus	-4.83	36	39	-9
	L	Medial orbital frontal cortex	-4.74	-18	48	-18
	L	Posterior orbital frontal cortex	-4.69	-30	21	-21
	R	Temporal pole	-4.60	45	21	-24
	R	Olfactory cortex	-4.59	15	15	-18
	R	Anterior orbital frontal cortex	-4.55	45	51	-15
	L	SupraMarginal gyrus	-4.53	-63	-51	24
	L	Inferior frontal gyrus	-4.46	-21	33	-12
	L	Supplementary motor area	-4.44	-6	-9	51
	R	Superior frontal gyrus	-4.36	9	51	0
	L	Rolandic operculum	-4.33	-54	9	0
	L	Putamen	-4.20	-15	15	-9

	L	Postcentral gyrus	-4.18	-60	-21	15
	L	Parahippocampal gyrus	-4.17	-15	6	-21
	R	Parahippocampal gyrus	-4.06	24	6	-33
	R	Middle temporal gyrus	-4.03	48	3	-21
	R	Heschl’s gyrus	-3.98	45	-24	12
	R	Anterior cingulate gyrus	-3.96	6	42	-3
	R	Amygdala	-3.85	27	3	-21
	L	Middle cingulate gyrus	-3.60	-6	-9	48
	R	Middle cingulate gyrus	-3.59	12	-12	42
	R	Putamen	-3.56	33	-3	-6
88	R	Superior temporal gyrus	-5.37	54	-6	-6
89	L	Insula	-5.33	-27	12	-15
39	L	Superior frontal gyrus	-5.01	-18	51	-12
12	L	Middle frontal gyrus	-4.53	-27	36	-12
135	R	Rolandic operculum	-4.30	48	-3	3
	R	SupraMarginal gyrus	-3.87	60	-21	18
112	R	Inferior temporal gyrus	-4.28	36	3	-42
	R	Fusiform gyrus	-4.06	39	-15	-30
11	R	Hippocampus	-4.11	30	-9	-18
16	L	Middle occipital gyrus	-3.40	-45	-72	6
10	L	Inferior occipital gyrus	-3.39	-33	-78	-12
11	L	Inferior temporal gyrus	-3.33	-36	12	-39

Seed ROI, contrast		***Voxel-level***		***MNI [mm]***		
cluster size	***Hemisphere***	***Brain region***	***Peak t-value***	***x***	***y***	***z***
Right BLA, PD<HC						
2930	R	Medial orbital frontal cortex	-6.73	15	57	-18
	R	Anterior orbital frontal cortex	-5.85	18	57	-18
	L	Anterior orbital frontal cortex	-5.68	-21	48	-18
	R	Insula	-5.66	27	21	-18
	L	Insula	-5.66	27	21	-18
	L	ventromedial prefrontal cortex	-5.57	3	33	-15
	R	Posterior orbital frontal cortex	-5.43	24	21	-18
	L	Gyrus rectus	-5.40	3	30	-18
	R	ventromedial prefrontal cortex	-5.38	3	36	-12
	L	Medial orbital frontal cortex	-5.32	-18	45	-18
	L	Superior temporal gyrus	-5.23	-48	0	-6
	R	Olfactory cortex	-5.10	9	15	-12
	R	Gyrus rectus	-5.00	6	39	-18
	R	Nucleus accumbens	-4.95	9	12	-12

	L	Olfactory cortex	-4.91	-24	6	-18
	L	Nucleus accumbens	-4.87	-12	12	-12
	L	Middle temporal gyrus	-4.86	-63	-15	-3
	R	Putamen	-4.85	33	0	-6
	L	Posterior orbital frontal cortex	-4.82	-21	9	-18
	R	Inferior frontal gyrus	-4.78	30	24	-12
	L	Temporal pole	-4.72	-54	12	-6
	R	Temporal pole	-4.65	48	6	-12
	L	Rolandic operculum	-4.63	-57	9	0
	R	Superior temporal gyrus	-4.41	54	-6	-9
	L	SupraMarginal gyrus	-4.13	-66	-30	18
	L	Inferior frontal gyrus	-4.09	-21	33	-12
	R	Rolandic operculum	-3.76	51	-3	3
	L	Putamen	-3.62	-12	9	-9
99	R	Superior frontal gyrus	-4.57	15	54	-12
79	L	Anterior cingulate gyrus	-4.50	0	36	-9
	L	Middle cingulate gyrus	-4.11	0	9	42
	R	Middle cingulate gyrus	-3.75	3	9	42
32	R	Middle frontal gyrus	-4.32	45	51	-12
142	R	Parahippocampal gyrus	-4.17	30	-9	-33
	R	Fusiform gyrus	-3.98	33	-9	-33
	L	Inferior temporal gyrus	-3.93	-33	-3	-45
24	L	Supplementary motor area	-4.11	-3	3	66
15	L	Parahippocampal gyrus	-4.10	-21	-33	-12
11	L	Amygdala	-4.10	-21	-33	-12
18	L	Postcentral gyrus	-4.10	-57	-21	15
32	R	Hippocampus	-3.83	36	-21	-18
12	L	Precentral gyrus	-3.44	-30	-24	54

Seed ROI, contrast		***Voxel-level***		***MNI [mm]***		
cluster size	***Hemisphere***	***Brain region***	***Peak t-value***	***x***	***y***	***z***
Left SFA, PD<HC						
1523	L	ventromedial prefrontal cortex	-5.65	3	30	-15
	L	Nucleus accumbens	-5.27	-9	15	-6
	L	Gyrus rectus	-5.13	0	33	-18
	R	Gyrus rectus	-4.99	9	48	-18
	R	Superior temporal gyrus	-4.94	54	-3	-9
	R	Olfactory cortex	-4.92	9	15	-12
	R	Temporal pole	-4.92	63	6	0
	R	Medial orbital frontal cortex	-4.80	18	30	-21
	R	ventromedial prefrontal cortex	-4.76	6	30	-15

	L	Anterior orbital frontal cortex	-4.74	-18	45	-15
	R	Middle temporal gyrus	-3.67	51	-66	9
	L	Olfactory cortex	-4.65	-21	6	-15
	L	Putamen	-4.63	-15	18	-9
	R	Insula	-4.42	39	-3	3
	R	Inferior frontal gyrus	-4.41	36	30	-9
	L	Inferior frontal gyrus	-4.34	-30	36	-6
	L	Medial orbital frontal cortex	-4.30	-18	45	-18
	R	Putamen	-4.30	27	3	-9
	R	Rolandic operculum	-4.29	48	-6	6
	R	Amygdala	-4.24	27	0	-12
	R	Heschl’s gyrus	-4.15	60	-3	6
	R	Anterior orbital frontal cortex	-3.98	24	39	-15
	R	Inferior temporal gyrus	-3.98	45	3	-39
725	L	Superior temporal gyrus	-5.40	-57	-12	-3
	L	Temporal pole	-5.34	-60	6	-3
	L	Rolandic operculum	-5.22	-57	9	0
	L	SupraMarginal gyrus	-4.05	-63	-24	15
	L	Insula	-3.98	-36	-18	3
51	R	Hippocampus	-5.24	36	-21	-18
	R	Parahippocampal gyrus	-4.55	33	-21	-21
107	L	Precentral gyrus	-5.10	-24	-24	75
	L	Postcentral gyrus	-4.85	-63	0	15
10	L	Middle temporal gyrus	-5.05	-60	-12	-3
175	L	Anterior cingulate gyrus	-4.70	-9	36	-9
	L	Supplementary motor area	-4.09	-6	-15	51
	L	Middle cingulate gyrus	-4.04	0	6	39
	R	Middle cingulate gyrus	-3.85	3	3	39
30	L	Lingual gyrus	-4.23	-18	-69	-9
34	L	Parahippocampal gyrus	-4.19	-27	0	-30
31	R	Fusiform gyrus	-4.17	39	-12	-33
	L	Fusiform gyrus	-4.11	-42	-48	-21
20	L	Inferior temporal gyrus	-4.06	-45	-48	-21
10	R	Precentral gyrus	-3.65	36	-12	48
10	L	Middle occipital gyrus	-3.50	-48	-78	3

Supplementary Table 3. Brain regions where FC contributes to all clinical variables (including demographic, motor, medication, brain volume, and NMS characteristics).

Seed ROI		*Voxel-level*		*MNI [mm]*		
cluster size	***Hemisphere***	***Brain region***	***BSR***	***x***	***y***	***z***
**Left CMA LV-1**						
23	L	Hippocampus	7.03	-27	-27	-12
652	R	Putamen	6.87	36	-3	-3
	R	Inferior frontal gyrus	6.58	30	27	-12
	R	Rolandic operculum	6.47	63	-6	12
	R	Superior Temporal Gyrus	5.74	51	-6	0
	R	Insula	5.29	39	-3	3
376	R	Supplementary motor area	6.81	3	0	63
	L	Supplementary motor area	6.50	0	-18	57
	L	Middle cingulate gyrus	5.59	-6	21	36
	R	Middle cingulate gyrus	4.92	6	6	39
296	L	Putamen	6.43	-30	3	-3
	L	Superior Temporal Gyrus	6.26	-57	-12	0
	L	Middle temporal gyrus	5.98	-51	-63	0
	L	Insula	5.85	-36	-15	0
	L	Inferior frontal gyrus	5.84	-30	33	-6
	L	Postcentral gyrus	5.65	-57	0	15
	L	Temporal pole	4.89	-33	18	-33
102	R	Orbital frontal cortex	6.42	27	30	-12
	L	ventromedial prefrontal cortex	5.64	-6	39	-9
	R	ventromedial prefrontal cortex	5.22	6	30	-15
	R	Gyrus rectus	5.13	21	15	-12
28	R	Temporal pole	5.95	48	12	-30
57	L	Inferior occipital gyrus	5.83	-36	-78	-9
52	R	Middle temporal gyrus	5.01	51	-51	-3
	R	Inferior temporal gyrus	4.32	48	-60	-9

Seed ROI		*Voxel-level*		*MNI [mm]*		
cluster size	***Hemisphere***	***Brain region***	***BSR***	***x***	***y***	***z***
**Left BLA LV-1**						
533	R	Temporal pole	7.62	42	12	-30
	R	Superior temporal gyrus	7.41	54	-6	-3
	R	Amygdala	6.82	30	-3	-27
	R	Inferior temporal gyrus	6.30	48	9	-36
	R	Rolandic operculum	5.79	63	-21	15
	R	Insula	5.68	39	-9	-6

111	L	Insula	7.14	-33	-18	6
	L	Olfactory cortex	5.94	-21	3	-18
	L	Putamen	5.13	-21	15	-9
	L	Inferior frontal gyrus	4.86	-24	30	-12
615	L	Middle temporal gyrus	6.70	-60	-15	0
	L	Superior temporal gyrus	6.65	-57	-12	-3
	L	Temporal pole	6.24	-54	6	-9
	L	Rolandic operculum	5.74	-57	0	12
	L	SupraMarginal gyrus	5.44	-57	-51	27
42	L	Supplementary motor area	6.48	-6	-12	48
427	R	Orbital frontal cortex	6.13	27	30	-15
	L	ventromedial prefrontal cortex	5.98	-6	30	-12
	R	ventromedial prefrontal cortex	5.97	3	27	-15
	R	Gyrus rectus	5.85	6	33	-18
	R	Inferior frontal gyrus	5.68	30	30	-12
	L	Orbital frontal cortex	5.42	-24	30	-15
	L	Medial orbital frontal cortex	5.11	21	27	-18
	L	Gyrus rectus	5.02	3	30	-18
40	L	Anterior cingulate gyrus	5.59	-6	36	-9
	L	Middle cingulate gyrus	5.17	-6	18	36
10	R	Hippocampus	5.57	27	-3	-27
14	R	Parahippocampal gyrus	5.55	33	-21	-21
15	L	Middle occipital gyrus	4.89	-48	-78	3

Seed ROI		***Voxel-level***		***MNI [mm]***		
cluster size	***Hemisphere***	***Brain region***	***BSR***	***x***	***y***	***z***
**Right BLA LV-1**						
139	R	Temporal pole	7.63	51	3	-9
	R	Inferior frontal gyrus	5.64	30	30	-12
574	L	Superior temporal gyrus	7.24	-60	-33	12
	L	Middle temporal gyrus	6.17	-60	-15	0
	L	Rolandic operculum	5.72	-57	3	9
	L	SupraMarginal gyrus	4.89	-60	-27	18
218	R	Superior temporal gyrus	7.21	51	0	-9
	R	Insula	5.91	51	-3	0
18	R	Parahippocampal gyrus	6.86	33	-21	-21
	R	Hippocampus	5.33	30	-6	-27
53	L	Amygdala	6.81	-27	-3	-24
	L	Olfactory cortex	4.46	-21	3	-18
27	R	Fusiform gyrus	6.71	36	-6	-30
38	L	Middle cingulate gyrus	6.49	-3	6	39
58	R	Orbital frontal cortex	5.61	30	24	-24
21	L	Supplementary motor area	5.38	-3	6	66

17	R	orbital frontal cortex	5.12	24	30	-18
24	R	Inferior temporal gyrus	4.78	42	-9	-33
88	L	ventromedial prefrontal cortex	4.63	-3	45	-12
	R	ventromedial prefrontal cortex	4.57	6	42	-9

Seed ROI		***Voxel-level***		***MNI [mm]***		
cluster size	***Hemisphere***	***Brain region***	***BSR***	***x***	***y***	***z***
**Left SFA LV-1**						
43	R	Hippocampus	7.29	30	-18	-18
	R	Parahippocampal gyrus	6.50	30	-24	-18
179	L	Superior temporal gyrus	7.23	-57	-15	0
	L	Temporal pole	7.12	-54	9	-9
	L	Middle temporal gyrus	6.74	-57	-15	-3
	L	Inferior temporal gyrus	5.83	-45	-48	-18
118	R	Inferior temporal gyrus	6.88	42	0	-42
	R	Temporal pole	6.58	42	15	-30
	R	Fusiform gyrus	5.33	36	-9	-33
19	L	Hippocampus	6.87	-24	-15	-21
	L	Parahippocampal gyrus	6.79	-30	0	-33
54	L	Supplementary motor area	6.73	0	-18	54
85	L	Postcentral gyrus	6.16	-33	-24	48
	L	Precentral gyrus	5.49	-21	-27	66
214	R	Superior temporal gyrus	5.99	54	-6	-9
38	R	Putamen	5.89	33	0	3
43	R	Amygdala	5.60	33	3	-21
43	L	Putamen	5.29	-18	15	-9
72	R	Gyrus rectus	4.75	6	27	-18
	L	ventromedial prefrontal cortex	4.52	0	36	-15

Supplementary Table 4. The roles of amygdala subregions in NMS.

	left CMA LV1	left BLA LV1	right BLA LV1	left SFA LV1
Gender	**-0.13**	-0.01	-0.04	**-0.16**
Age	**-0.40**	**-0.34**	**-0.37**	**-0.40**
Education	**0.10**	**0.10**	**0.17**	**0.12**
Disease duration	-0.06	-0.08	-0.10	**-0.08**
UPDRS III	**-0.09**	**-0.20**	**-0.22**	**-0.16**
H-Y stage	**-0.15**	**-0.21**	**-0.21**	**-0.24**
LEDD	**-0.08**	**-0.10**	**-0.13**	**-0.07**

L-CM volume	**-0.18**	**-0.18**	**-0.18**	**-0.13**
L-LB volume	**-0.16**	**-0.18**	**-0.16**	**-0.13**
L-SF volume	**-0.17**	**-0.16**	**-0.17**	**-0.15**
R-CM volume	**-0.12**	**-0.11**	-0.06	-0.07
R-LB volume	-0.08	-0.08	-0.05	**-0.09**
R-SF volume	**-0.11**	**-0.09**	-0.08	**-0.08**
TIV	**0.11**	**0.16**	**0.12**	0.04
GM volume	**0.24**	**0.31**	**0.30**	**0.19**
GDS-15	**-0.12**	**-0.12**	**-0.18**	**-0.07**
UPSIT	**0.22**	**0.19**	**0.21**	**0.27**
MoCA	**0.28**	**0.24**	**0.26**	**0.24**
RBD-SQ	**-0.13**	**-0.11**	**-0.11**	**-0.10**
ESS	-0.03	-0.06	**-0.10**	**-0.08**
QUIP	-0.01	0.02	0.08	0.02
STAI	**-0.24**	**-0.29**	**-0.23**	**-0.25**
Apathy	**-0.21**	**-0.27**	**-0.25**	**-0.28**
Hallucinations	**-0.15**	**-0.09**	**-0.09**	**-0.12**
Pain	**-0.14**	**-0.12**	**-0.18**	**-0.14**
Urinary disorder	**-0.24**	**-0.14**	**-0.17**	**-0.18**
Constipation	**-0.23**	**-0.22**	**-0.14**	**-0.27**
OH	**-0.08**	**-0.10**	-0.04	**-0.09**
Fatigue	**-0.18**	**-0.20**	**-0.09**	**-0.22**
Drooling	**-0.16**	**-0.15**	**-0.15**	**-0.16**
Swallowing	**-0.14**	-0.06	**-0.08**	**-0.10**

Bold values indicate the weights are statistically significant.

Supplementary Table 5. Brain regions where FC contributes to NMS (have regressed demographic, motor, medication, and brain volume characteristics).

Seed ROI		***Voxel-level***		***MNI [mm]***		
cluster size	***Hemisphere***	***Brain region***	***BSR***	***x***	***y***	***z***
**Left CMA LV-1**						
39	R	Temporal pole	7.58	51	6	-18
74	L	Inferior frontal gyrus	6.79	-45	24	-9
	L	Temporal pole	6.69	-45	21	-24

68	L	Middle temporal gyrus	6.19	-60	-18	-9
40	R	Amygdala	6.04	27	0	-12
	R	Putamen	5.73	24	6	-9
30	L	Nucleus accumbens	6.00	-6	15	-3
	R	Middle temporal gyrus	5.22	54	-57	6
137	L	Supplementary motor area	5.86	-6	12	69
	R	Supplementary motor area	4.49	3	-12	57
	L	Middle cingulate gyrus	4.34	-6	-15	45
10	L	Hippocampus	4.41	-21	-30	-9

Seed ROI		***Voxel-level***		***MNI [mm]***		
cluster size	***Hemisphere***	***Brain region***	***BSR***	***x***	***y***	***z***
**Left BLA LV-1**						
257	L	Superior temporal gyrus	6.96	-51	-9	6
	L	Middle temporal gyrus	6.58	-57	-51	9
	L	Temporal pole	6.13	-54	12	-6
	L	Rolandic operculum	4.53	-57	3	3
	L	Inferior frontal gyrus	4.31	-45	24	-12
92	R	Amygdala	6.61	24	3	-21
	R	Temporal pole	6.03	27	6	-21
	R	Parahippocampal gyrus	5.64	27	9	-24
	R	Inferior temporal gyrus	5.14	42	3	-45
85	R	Insula	5.69	48	-3	-3
	R	Superior temporal gyrus	5.08	57	-12	6
81	L	Supplementary motor area	5.67	-6	-3	51
	L	Middle cingulate gyrus	5.12	-3	-3	48
	R	Middle cingulate gyrus	4.93	12	-12	42
7	R	Putamen	5.51	33	0	-6
17	L	Insula	5.22	-36	0	-9
21	R	SupraMarginal gyrus	4.93	63	-24	18
22	L	SupraMarginal gyrus	4.68	-63	-42	24

Seed ROI		***Voxel-level***		***MNI [mm]***		
cluster size	***Hemisphere***	***Brain region***	***BSR***	***x***	***y***	***z***
**Right BLA LV-1**						
348	L	Temporal pole	7.43	-57	6	0
	L	Superior temporal gyrus	6.01	-45	-42	18
	L	Insula	6.01	-36	0	-9
	L	Middle temporal gyrus	5.84	-54	6	-18
	L	SupraMarginal gyrus	5.21	-60	-42	24
	L	Inferior frontal gyrus	4.77	-45	24	-12
146	R	Putamen	7.25	33	-3	-6

	R	Insula	6.61	51	9	-6
	R	Superior temporal gyrus	6.13	63	-15	-6
	R	Temporal pole	6.05	51	6	-9
71	L	Middle cingulate gyrus	6.60	-3	3	42
	L	Supplementary motor area	6.25	-9	6	69
348	R	Middle cingulate gyrus	6.00	6	15	33
**Right BLA LV-2**						
202	R	Ventromedial prefrontal cortex	7.26	9	45	-6
	L	Ventromedial prefrontal cortex	6.28	-3	48	-12
	R	Gyrus rectus	5.81	6	45	-18
	L	Gyrus rectus	4.56	0	45	-18
	L	Anterior orbital gyrus	4.83	-27	42	-15
10	R	Medial orbital gyrus	4.62	21	39	-21
10	R	Anterior orbital gyrus	4.03	36	60	-15

Seed ROI		***Voxel-level***		***MNI [mm]***		
cluster size	***Hemisphere***	***Brain region***	***BSR***	***x***	***y***	***z***
**Left SFA LV-1**						
45	L	Supplementary motor area	6.81	-9	6	63
16	R	Amygdala	6.48	24	0	-18
119	L	Middle temporal gyrus	6.19	-51	9	-24
	L	Temporal pole	5.99	-51	9	-21
	L	Superior temporal gyrus	5.88	-57	0	-9
25	R	Inferior temporal gyrus	5.83	42	3	-45
13	R	Temporal pole	5.63	45	18	-33
32	R	Superior temporal gyrus	4.93	57	-12	6
10	R	Hippocampus	4.33	33	-18	-18
**Left SFA LV-2**						
34	L	Nucleus accumbens	7.06	-12	18	-6
	L	Putamen	5.91	-18	18	-9
133	L	Ventromedial prefrontal cortex	6.97	-12	33	-12
	R	Ventromedial prefrontal cortex	5.94	12	42	-9
	L	Middle cingulate gyrus	4.86	0	18	36
63	L	Medial orbital gyrus	6.71	-18	39	-18
	L	Anterior orbital gyrus	6.61	-18	39	-15
	L	Gyrus rectus	6.30	-12	42	-18
34	R	Gyrus rectus	4.73	9	36	-18

Supplementary Table 6. The roles of amygdala subregions in NMS.

	left CMA	left BLA	right BLA	right BLA	left SFA	left SFA

	LV1	LV1	LV1	LV2	LV1	LV2
GDS-15	**-0.35**	**-0.32**	**-0.46**	**0.15**	**-0.30**	**0.21**
UPSIT	**0.25**	**0.36**	**0.37**	0.05	**0.41**	**0.18**
MoCA	**0.40**	**0.25**	**0.15**	**-0.12**	**0.20**	0.03
RBD-SQ	**-0.28**	**-0.18**	**-0.28**	**0.45**	**-0.36**	**0.34**
ESS	0.01	-0.02	0.04	-0.02	0.00	**-0.15**
QUIP	-0.05	0.09	**0.25**	**0.34**	**0.14**	-0.04
STAI	**-0.34**	**-0.50**	**-0.27**	**0.17**	**-0.34**	**-0.19**
Apathy	**-0.29**	**-0.44**	**-0.35**	**0.13**	**-0.34**	**-0.21**
Hallucinations	**-0.11**	**0.18**	**0.21**	**0.16**	**-0.15**	**0.24**
Pain	**-0.18**	**-0.12**	**-0.15**	-0.07	**-0.15**	**-0.26**
Urinary disorder	**-0.31**	0.03	-0.06	**0.31**	**-0.21**	**0.32**
Constipation	**-0.20**	-0.05	**0.17**	**0.31**	**-0.31**	-0.01
OH	0.09	**0.16**	**0.26**	**0.35**	0.00	**0.36**
Fatigue	-0.11	**-0.15**	**0.17**	**0.23**	**-0.14**	**-0.36**
Drooling	**-0.24**	-0.07	0.01	**0.16**	**-0.11**	**-0.18**
Swallowing	**-0.21**	-0.11	**-0.12**	**0.34**	**-0.19**	**0.32**

Bold values indicate the weights are statistically significant.

Supplementary Table 7. One-way ANOVA tests on tissue volumes in raw and harmonized structural data.

	**Raw data**	**Harmonized data**
	**F value (P value)**	**F value (P value)**
Total intracranial volume	0.270 (0.965)	0.293 (0.956)
Grey matter volume	0.310 (0.949)	0.068 (1.000)
White matter volume	0.468 (0.857)	0.379 (0.914)
Cerebrospinal fluid volume	0.772 (0.611)	0.832 (0.562)
Left CMA volume	1.369 (0.221)	0.789 (0.597)
Right CMA volume	1.365 (0.223)	0.424 (0.887)

Left BLA volume	0.601 (0.755)	0.324 (0.942)
Right BLA volume	0.460 (0.863)	0.166 (0.992)
Left SFA volume	0.708 (0.665)	0.269 (0.965)
Right SFA volume	1.482 (0.176)	0.307 (0.950)
