# Supplementary material for: Common and distinct roles of amygdala subregional functional connectivity in non-motor symptoms of Parkinson’s disease
Source: NPJ Parkinsons Dis. 2023 Feb 17;9:28. doi: 10.1038/s41531-023-00469-1 (PMC9938150; doi:10.1038/s41531-023-00469-1)
Supplement: Supplementary file 2 — Reporting Summary [file 41531_2023_469_MOESM2_ESM.pdf]

nature portfolio

Corresponding author(s): Mingrui Xia and Tao Wu

Last updated by author(s): Jan 25, 2023

Reporting Summary

Nature Portfolio wishes to improve the reproducibility of the work that we publish. This form provides structure for consistency and transparency in reporting. For further information on Nature Portfolio policies, see our Editorial Policies and the Editorial Policy Checklist.

Statistics

For all statistical analyses, confirm that the following items are present in the figure legend, table legend, main text, or Methods section.

n/a	Confirmed	
☐	☒	The exact sample size (n) for each experimental group/condition, given as a discrete number and unit of measurement
☐	☒	A statement on whether measurements were taken from distinct samples or whether the same sample was measured repeatedly
☐	☒	The statistical test(s) used AND whether they are one- or two-sided<br>*Only common tests should be described solely by name; describe more complex techniques in the Methods section.*
☐	☒	A description of all covariates tested
☐	☒	A description of any assumptions or corrections, such as tests of normality and adjustment for multiple comparisons
☐	☒	A full description of the statistical parameters including central tendency (e.g. means) or other basic estimates (e.g. regression coefficient) AND variation (e.g. standard deviation) or associated estimates of uncertainty (e.g. confidence intervals)
☐	☒	For null hypothesis testing, the test statistic (e.g. F, t, r) with confidence intervals, effect sizes, degrees of freedom and P value noted<br>*Give P values as exact values whenever suitable.*
☐	☒	For Bayesian analysis, information on the choice of priors and Markov chain Monte Carlo settings
☐	☒	For hierarchical and complex designs, identification of the appropriate level for tests and full reporting of outcomes
☐	☒	Estimates of effect sizes (e.g. Cohen's d, Pearson's r), indicating how they were calculated

Our web collection on statistics for biologists contains articles on many of the points above.

Software and code

Policy information about availability of computer code

Data collection	Parkinson's Progression Markers Initiative
Data analysis	DPABI (v6.0), FSL-FAST (5.0), FSL-FIRST (v5.0), ANTs (v2.0), SPM12, SPSS 22.0, BrainNet Viewer (v1.7), myPLS (https://github.com/danizoeller/myPLS).

For manuscripts utilizing custom algorithms or software that are central to the research but not yet described in published literature, software must be made available to editors and reviewers. We strongly encourage code deposition in a community repository (e.g. GitHub). See the Nature Portfolio guidelines for submitting code & software for further information.

Data

Policy information about availability of data

All manuscripts must include a data availability statement. This statement should provide the following information, where applicable:

- Accession codes, unique identifiers, or web links for publicly available datasets
- A description of any restrictions on data availability
- For clinical datasets or third party data, please ensure that the statement adheres to our policy

Human research participants

Policy information about studies involving human research participants and Sex and Gender in Research.

Reporting on sex and gender	In total, 115 patients with PD (male/female: 59/56) and 78 healthy controls (HCs) (male/female: 36/42) were included in this study. No significant differences were observed regarding sex between the HC and PD groups.
Population characteristics	In total, 115 patients with PD and 78 healthy controls (HCs) were included in this study. No significant differences were observed regarding age (59.50 ± 9.4 vs 60.68 ± 8.8 years), sex (male/female: 59/56 vs 36/42), or education level (12 [9,16] vs 12 [9,16] years) between the PD and HC groups.
Recruitment	We enrolled 193 participants (PD: 115, HC: 78) from two independent cohorts. Specifically, 157 participants (PD: 86, HC: 71) from cohort 1 were recruited from June 2017 to May 2021 from the Department of Neurology, Xuanwu Hospital Capital Medical University. In cohort 2, 36 participants (PD: 29, HC: 7) were obtained from the Parkinson's Progression Markers Initiative (PPMI) database (https://www.ppmi-info.org/). The HCs were recruited from the community.
Ethics oversight	This study was approved by the Ethics Committee of Xuanwu Hospital of Capital Medical University.

Note that full information on the approval of the study protocol must also be provided in the manuscript.

Field-specific reporting

Please select the one below that is the best fit for your research. If you are not sure, read the appropriate sections before making your selection.

☒ Life sciences ☐ Behavioural & social sciences ☐ Ecological, evolutionary & environmental sciences

For a reference copy of the document with all sections, see nature.com/documents/nr-reporting-summary-flat.pdf

Life sciences study design

All studies must disclose on these points even when the disclosure is negative.

Sample size	We enrolled 193 participants (PD: 115, HC: 78) from two independent cohorts. Specifically, 157 participants (PD: 86, HC: 71) from cohort 1 were recruited from June 2017 to May 2021 from the Department of Neurology, Xuanwu Hospital Capital Medical University. In cohort 2, 36 participants (PD: 29, HC: 7) were obtained from the Parkinson's Progression Markers Initiative (PPMI) database. The sample size is 187 and is sufficient.
Data exclusions	Twenty patients with PD and 14 HCs were excluded due to excessive head movements; 21 HCs were excluded due to cognitive impairment, anxiety, or sleep disorders.
Replication	We validated our results by considering the following aspects. First, to avoid subregion selection bias due to separate PLS modeling of the four subregions, we used data from all four subregions to construct one PLS model to validate the association between FC changes in the amygdala and NMS (Supplementary Figure 4). Second, we used one-way ANOVA tests on both raw data and harmonized data, with multi-sites as the factor, to examine whether our findings were influenced by specific sites. Significant main effects were observed on raw FC maps but not on harmonized FC maps (Supplementary Figure 5 and Supplementary Table 7), indicating the site effect was corrected in the data. Third, to further examine whether the site effect had impact on our results, we removed the data from cohort 2 and performed FC analysis using only the data from cohort 1, and the validated results were consistent with the results of multicenter data (Supplementary Figure 6 and Supplementary Figure 7).
Randomization	For all patients or healthy controls who met the inclusion criteria, we have included them.
Blinding	Our study did not set up an intervention, there were only two groups, the PD group and the control group. Therefore, no blinding was required.

Behavioural & social sciences study design

All studies must disclose on these points even when the disclosure is negative.

Study description	
Research sample	
Sampling strategy	

Data collection

Timing

Data exclusions

Non-participation

Randomization

Ecological, evolutionary & environmental sciences study design

All studies must disclose on these points even when the disclosure is negative.

Study description

Research sample

Sampling strategy

Data collection

Timing and spatial scale

Data exclusions

Reproducibility

Randomization

Blinding

Did the study involve field work? ☐ Yes ☐ No

Field work, collection and transport

Field conditions

Location

Access & import/export

Disturbance

Reporting for specific materials, systems and methods

We require information from authors about some types of materials, experimental systems and methods used in many studies. Here, indicate whether each material, system or method listed is relevant to your study. If you are not sure if a list item applies to your research, read the appropriate section before selecting a response.

Materials & experimental systems

n/a	Involved in the study
☒	☐ Antibodies
☒	☐ Eukaryotic cell lines
☒	☐ Palaeontology and archaeology
☒	☐ Animals and other organisms
☒	☐ Clinical data
☒	☐ Dual use research of concern

Methods

n/a	Involved in the study
☒	☐ ChIP-seq
☒	☐ Flow cytometry
☐	☒ MRI-based neuroimaging

Antibodies

Antibodies used

Validation

Eukaryotic cell lines

Policy information about cell lines and Sex and Gender in Research

Cell line source(s)

Authentication

Mycoplasma contamination

Commonly misidentified lines
(See ICLAC register)

Palaeontology and Archaeology

Specimen provenance

Specimen deposition

Dating methods

☐ Tick this box to confirm that the raw and calibrated dates are available in the paper or in Supplementary Information.

Ethics oversight

Note that full information on the approval of the study protocol must also be provided in the manuscript.

Animals and other research organisms

Policy information about studies involving animals; ARRIVE guidelines recommended for reporting animal research, and Sex and Gender in Research

Laboratory animals

Wild animals

Reporting on sex

Field-collected samples

Ethics oversight

Note that full information on the approval of the study protocol must also be provided in the manuscript.

Clinical data

Policy information about clinical studies

All manuscripts should comply with the ICMJE guidelines for publication of clinical research and a completed CONSORT checklist must be included with all submissions.

Clinical trial registration

Study protocol

Data collection

Outcomes

Dual use research of concern

Policy information about dual use research of concern

Hazards

Could the accidental, deliberate or reckless misuse of agents or technologies generated in the work, or the application of information presented in the manuscript, pose a threat to:

No	Yes	
☐	☐	Public health
☐	☐	National security
☐	☐	Crops and/or livestock
☐	☐	Ecosystems
☐	☐	Any other significant area

Experiments of concern

Does the work involve any of these experiments of concern:

No	Yes	
☐	☐	Demonstrate how to render a vaccine ineffective
☐	☐	Confer resistance to therapeutically useful antibiotics or antiviral agents
☐	☐	Enhance the virulence of a pathogen or render a nonpathogen virulent
☐	☐	Increase transmissibility of a pathogen
☐	☐	Alter the host range of a pathogen
☐	☐	Enable evasion of diagnostic/detection modalities
☐	☐	Enable the weaponization of a biological agent or toxin
☐	☐	Any other potentially harmful combination of experiments and agents

ChIP-seq

Data deposition

☐ Confirm that both raw and final processed data have been deposited in a public database such as GEO.

☐ Confirm that you have deposited or provided access to graph files (e.g. BED files) for the called peaks.

Data access links
May remain private before publication.

Files in database submission

Genome browser session
(e.g. UCSC)

Methodology

Replicates

Sequencing depth

Antibodies

Peak calling parameters

Data quality

Software

Flow Cytometry

Plots

Confirm that:

☐ The axis labels state the marker and fluorochrome used (e.g. CD4-FITC).

☐ The axis scales are clearly visible. Include numbers along axes only for bottom left plot of group (a 'group' is an analysis of identical markers).

☐ All plots are contour plots with outliers or pseudocolor plots.

☐ A numerical value for number of cells or percentage (with statistics) is provided.

Methodology

Sample preparation	
Instrument	
Software	
Cell population abundance	
Gating strategy	

☐ Tick this box to confirm that a figure exemplifying the gating strategy is provided in the Supplementary Information.

Magnetic resonance imaging

Experimental design

Design type	resting state
Design specifications	The MRI data in cohort 1 were acquired using a Siemens Magnetom Skyra 3T scanner (Erlangen, Germany). Rs-fMRI images were obtained using a single-shot spin-echo echo-planar imaging (SE-EPI) sequence. Rs-fMRI images were obtained with the following parameters: repetition time (TR), 2000 ms; echo time (TE), 30 ms; field of view (FOV), 22 × 22 cm2; flip angle, 90°; voxel size, 3.4 × 3.4 × 3 mm2; 35 slices, no gap; 176 repetitions; scanning time, 5 min 52 s. T1-weighted anatomic images were scanned using a magnetization-prepared 3D rapid gradient echo (MPRAGE) sequence. The image parameters were as follows: TR/TE/flip angle, 2530 ms/2.98 ms/7°; FOV, 25.6 × 25.6 cm2; voxel size 1 × 1 × 1 mm2; 192 slices, no gap. The acquisition time was 5 min 13 s. MRI data from the PPMI dataset were scanned with Siemens Trio 3T scanners, using the same acquisition protocol to ensure data standardization. In brief, rs-fMRI images were obtained using the SE-EPI sequence. The imaging parameters were as follows: TR/TE/flip angle, 2400 ms/25 ms/80°; FOV, 22 × 22 cm2; voxel size, 3.3 × 3.3 × 3.3 mm2; 40 slices, no gap. 210 repetitions; scanning time, 8 min 24 s.
Behavioral performance measures	None

Acquisition

Imaging type(s)	functional and structural
Field strength	3.0T
Sequence & imaging parameters	The MRI data in cohort 1 were acquired using a Siemens Magnetom Skyra 3T scanner (Erlangen, Germany). Rs-fMRI images were obtained using a single-shot spin-echo echo-planar imaging (SE-EPI) sequence. Rs-fMRI images were obtained with the following parameters: repetition time (TR), 2000 ms; echo time (TE), 30 ms; field of view (FOV), 22 × 22 cm2; flip angle, 90°; voxel size, 3.4 × 3.4 × 3 mm2; 35 slices, no gap; 176 repetitions; scanning time, 5 min 52 s. T1-weighted anatomic images were scanned using a magnetization-prepared 3D rapid gradient echo (MPRAGE) sequence. The image parameters were as follows: TR/TE/flip angle, 2530 ms/2.98 ms/7°; FOV, 25.6 × 25.6 cm2; voxel size 1 × 1 × 1 mm2; 192 slices, no gap. The acquisition time was 5 min 13 s. MRI data from the PPMI dataset were scanned with Siemens Trio 3T scanners, using the same acquisition protocol to ensure data standardization. In brief, rs-fMRI images were obtained using the SE-EPI sequence. The imaging parameters were as follows: TR/TE/flip angle, 2400 ms/25 ms/80°; FOV, 22 × 22 cm2; voxel size, 3.3 × 3.3 × 3.3 mm2; 40 slices, no gap. 210 repetitions; scanning time, 8 min 24 s. T1-weighted anatomical images were scanned using an MPRAGE sequence. The imaging parameters were as follows: TR/TE/flip angle, 2300 ms/3 ms/9°; FOV, 25.6 × 24 cm2; voxel size, 1 × 1 × 1 mm2; 170 slices, no gap.
Area of acquisition	

Diffusion MRI ☐ Used ☒ Not used

Preprocessing

Preprocessing software	DPABI (v6.0). Imaging data were preprocessed and analyzed using DPABI (v6.0; http://www.rfmri.org/dpabi), including removal of the first ten volumes, slice timing correction, head motion correction, tissue segmentation, spatial normalization into Montreal Neurological Institute (MNI) space and resampling into 3-mm isotropic voxels, spatial smoothing with a full width at half maximum of 6 mm Gaussian kernel, and temporal bandpass filtering at 0.01–0.08 Hz.
Normalization	We use DARTEL to perform normalization. Specifically, the structural image was first normalized to the functional image space, then the structural image was segmented into gray matter white matter cerebrospinal fluid, and also transformation matrices were generated, a group template was subsequently generated based on all subjects, and then all structural images were normalized to this group template, and finally normalized to the MNI space.
Normalization template	ICBM152
Noise and artifact removal	Participants were excluded if their mean framewise displacement (FD) was > 0.3 mm, or if the translational/rotational movements were > 3 mm or 3°. During image preprocessing, signals from head movement, white matter, and cerebrospinal fluid were regressed off as covariates.
Volume censoring	DPABI

Statistical modeling & inference

Model type and settings	SPM12 was used to calculate within- and between-groups differences in FC. For the FC maps of each amygdala subregion, we first applied one-sample t-tests to the PD and HC groups to determine the connectivity profile of each subregion. The significant level for the within-group test was set at a family-wise error (FWE) corrected P < 0.05 at voxel-level with a cluster size ≥ 10 voxels. An FC mask was then generated as the union of the within-group results from both groups for each subregion. Between-group differences in FC maps for each amygdala subregion were then measured using the general linear model within this mask, with age, sex, and education as covariates, and the significant level was FDR corrected P < 0.05 at the voxel level with a cluster size ≥ 10 voxels.
Effect(s) tested	NONE.

Specify type of analysis: ☐ Whole brain ☒ ROI-based ☐ Both

Statistic type for inference (See Eklund et al. 2016)	voxel-wise
Correction	One-sample t-tests to the PD and HC groups to determine the connectivity profile of each subregion. The significant level for the within-group test was set at a family-wise error (FWE) corrected P < 0.05 at voxel-level with a cluster size ≥ 10 voxels. Between-group differences in FC maps for each amygdala subregion were then measured using the general linear model within this mask, with age, sex, and education as covariates, and the significant level was FDR corrected P < 0.05 at the voxel level with a cluster size ≥ 10 voxels.

Models & analysis

n/a	Involved in the study
☐	☒ Functional and/or effective connectivity
☒	☐ Graph analysis
☒	☐ Multivariate modeling or predictive analysis

Functional and/or effective connectivity	Pearson's correlation
Graph analysis	
Multivariate modeling and predictive analysis	
